# Insights into soft short circuit-based degradation of lithium metal batteries†

**DOI:** 10.1039/d3fd00101f

**Published:** 2023-06-19

**Authors:** Svetlana Menkin, Jana B. Fritzke, Rebecca Larner, Cas de Leeuw, Yoonseong Choi, Anna B. Gunnarsdóttir, Clare P. Grey

**Affiliations:** a Yusuf Hamied Department of Chemistry, University of Cambridge Lensfield Road Cambridge CB2 1EW UK cpg27@cam.ac.uk sm2383@cam.ac.uk; b The Faraday Institution, Quad One, Harwell Science and Innovation Campus Didcot UK; c Faculty of Industrial Engineering, Mechanical Engineering and Computer Science, University of Iceland Reykjavík Iceland

## Abstract

The demand for electric vehicles with extended ranges has created a renaissance of interest in replacing the common metal-ion with higher energy-density metal-anode batteries. However, the potential battery safety issues associated with lithium metal must be addressed to enable lithium metal battery chemistries. A considerable performance gap between lithium (Li) symmetric cells and practical Li batteries motivated us to explore the correlation between the shape of voltage traces and degradation. We coupled impedance spectroscopy and *operando* NMR and used the new approach to show that transient (*i.e.*, soft) shorts form in realistic conditions for battery applications; however, they are typically overlooked, as their electrochemical signatures are often not distinct. The typical rectangular-shaped voltage trace, widely considered ideal, was proven, under the conditions studied here, to be a result of soft shorts. Recoverable soft-shorted cells were demonstrated during a symmetric cell polarisation experiment, defining a new type of critical current density: the current density at which the soft shorts are not reversible. Moreover, we demonstrated that soft shorts, detected *via* electrochemical impedance spectroscopy (EIS) and validated *via operando* NMR, are predictive towards the formation of hard shorts, showing the potential use of EIS as a relatively low-cost and non-destructive method for early detection of catastrophic shorts and battery failure while demonstrating the strength of *operando* NMR as a research tool for metal plating in lithium batteries.

## Abstract 2

A considerable performance gap between Li symmetric cells and practical Li batteries motivated us to explore the correlation between the shape of voltage traces and degradation.

## Graphical abstract

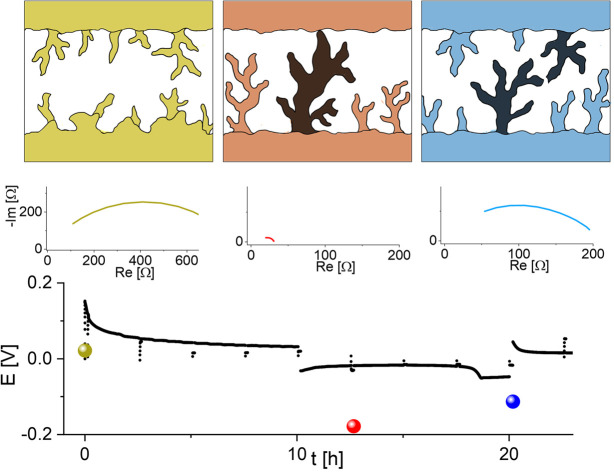

## Introduction

The demand for electric vehicles has created a renaissance of interest in replacing graphite with a higher energy-density lithium (Li) metal anode. Li metal has the highest volumetric and gravimetric energy density of all negative electrodes; however, it suffers from capacity fade and potential safety issues. The uneven electrodeposition of Li results in dendrite formation and potentially hazardous situations such as cell short-circuiting and thermal runaway. Although lithium plating has been studied widely, a better understanding of the short-circuiting mechanisms is required.

The most common way to study new electrolytes, additives, and artificial interfaces has been to use Li symmetric cells, *i.e.*, Li–Li cells where Li is plated and stripped on Li metal anodes over many cycles.^1–3^ However, a considerable performance gap is observed between Li symmetric cells and asymmetric, *i.e.*, metal (metal lithium *vs.* lithium-ion cathode) or anode-free (copper current collector *vs.* lithium-ion cathode) batteries.^4^ Developing a reliable testing procedure for lithium metal cells is critical for realising emerging “beyond Li-ion” battery technologies.

The overpotential (*η*_t_) that develops during plating and stripping of a non-passivated metal (*e.g.*, copper) originates from the ohmic resistance (*η*_ir_) of the system, mainly attributed to the current collectors and transport through the electrolyte, and the charge-transfer resistance at the electrode surface (that includes the overpotential required to overcome the activation energy barrier of metal nucleation (*η*_ac_)). A concentration polarisation (*η*_c_), established during plating, due to metal ion depletion near the plated electrode, also adds to *η*_t_. Due to the dynamic character of the overpotential that results from the formed concentration gradient, plating voltage traces are not flat but rather inherently curved.

Passivated metals (*e.g.*, Li) typically plate in organic electrolytes. Since the typical battery electrolyte's reduction potential is higher than that for alkali metal ion reduction, a passivating layer termed the solid electrolyte interphase (SEI) is formed upon the contact of the metal with the electrolyte by precipitation of electrolyte degradation products. The morphology and Li plating efficiency has been shown to correlate with the transport properties and formation kinetics of the SEI, which influences the current distribution over the electrode surface.^5,6^ The SEI introduces an additional overpotential (*η*_SEI_) due to hindered Li^+^ transport through the interphase layer and potentially, the ion concentration polarisation at the SEI–electrolyte interphase. The overall overpotential in these systems can be summarised as:

1 *η*_t_ = *η*_ir_ + *η*_SEI_ + *η*_ac_

Prolonged galvanostatic cycling of symmetric cells has been regarded as a key metric indicating the viability of a particular metal anode–electrolyte system. The voltage traces – *i.e.*, changes in overpotential, during plating and stripping yield information on the nucleation and growth of metal microstructures.^1,2^ A sharp nucleation peak at the beginning of plating has often been associated with dendritic Li morphology, and a rectangular-shaped voltage profile with a minimal overpotential is often reported as ideal.^2^ Prolonged cycling can, however, lead to short circuits, *i.e.*, an abnormal electrical circuit that allows current to travel along an unintended path with very low resistance. A short circuit in a battery might result in an exceedingly high current, which will cause local heating and potential accelerated thermal runaway, a cell failure that might lead to significant safety issues, *i.e.*, battery fire. Often the short-circuits are proceeded by an accumulation of dead lithium, which causes rapid cell degradation due to electrolyte consumption and increases the risk for short circuits. Degradation caused by electrolyte depletion is especially critical in commercial pouch cells, where the electrolyte per active material ratio is significantly lower than in standard coin cells generally implemented in lab research.

**Fig. 1:**
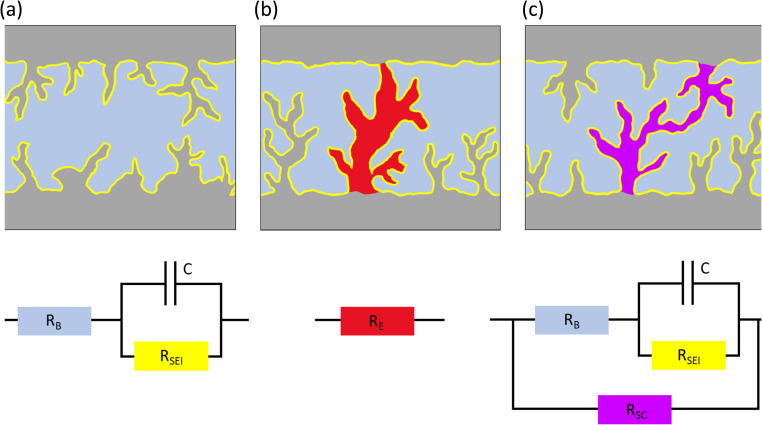
Illustrations and equivalent circuits for charge transport through electrode–electrolyte interface (purely ion transport) (left; (a)), hard short circuit (electron transport) (middle; (b)), soft short circuit (mixed electron and ion transport) (right; (c)).

Electrochemical impedance spectroscopy (EIS) is a common tool to monitor electrochemical dynamics. EIS measurements of symmetric cells typically result in Nyquist plots dominated by at least one semi-circle at medium frequencies, fitted with an equivalent circuit that consists of at least one resistor (*R*) in series with an RC unit (a capacitor and a resistor in parallel) (Fig. 1a). The charge transfer resistance (*R*_ct_) through the SEI is the dominant contributor to the impedance of Li symmetric cells. A decrease in impedance of symmetric cells is often observed and can arise from an increase in electrode surface area (due to roughening from plating), improved transport through the interphase due to maturing of the SEI, improved wetting of the electrode or increased temperatures. Typically, the *R*_ct_ will decline during the initial cycling and then grow due to the degradation, *e.g.*, accumulation of SEI. However, a short circuit in symmetric cells results in a pure resistor behaviour with a typical EIS Nyquist spectrum showing a linear response, without an imaginary loop, at the negative imaginary impedance values (Fig. 1b).

The shorts described above are generally referred to as hard, where the unintended electronic path is persistent and has a resistance comparable to a metal wire. Soft shorts are generally formed in less harsh conditions than hard shorts, resulting from small localized electrical connections between two electrodes allowing the co-existence of direct electrons transfer and an interfacial reaction, *e.g.*, in equivalent circuit terminology, a charge transfer resistor (*R*_ct_) is in parallel with another (less) resistive element, defined as a short circuit resistor (*R*_sc_), allowing electrons to pass through the short-circuiting wire, while also being consumed by SEI formation (Fig. 1c). When the magnitude of *R*_ct_ is comparable to *R*_sc_, the system can deliver symmetric cell performance that resembles a good battery behaviour, with low overpotentials.^7^ Illustrations and equivalent circuits for a non-shorted symmetric, hard shorted and soft shorted cell, are depicted in Fig. 1.

Although soft shorts were identified in the early nineties as a major safety issue, studies of their detection and prevention are scarce. Early publications by Moli Energy (1993)^8^ identified soft shorts as a potential failure route for Li metal batteries, however publications on the detection of soft shorts in Li metal full cells and symmetric cells are scarce. Albertus *et al.*^4^ demonstrated that Li symmetric cell performance is often not addressing realistic goals for Li metal batteries and suggested that reports of stable lithium metal cycling in Li symmetric cells are often the result of soft shorts. Li *et al.*^7^ showed for symmetric zinc cells that when the typical peaking behaviour in the voltage trace is replaced with a rectangular curve, the metal symmetric cell is, in fact, partially short-circuited.

We hypothesise that the rectangular-shaped voltage traces often portrayed in literature have a significant electronic transport component, *i.e.* they can represent soft shorts. Purely rectangular voltage profiles are an indication of the absence of concentration polarization. In this work, we start by explaining the typically observed voltage traces in Li symmetric cells and then go on to test our hypotheses. We show that soft short circuits occur in Li symmetric cells under realistic cycling conditions. We demonstrate that while only hard short circuits can be inferred solely by the shape of the voltage profile, soft shorts can be measured with customised *in situ* galvanostatic impedance spectroscopy (EIS). Soft shorts formation was validated using *operando* nuclear magnetic resonance (NMR).

The effect of electrolyte composition on battery performance is widely studied (albeit significantly less than electrode materials). Numerous studies in the literature identified salts (*e.g.*, LiTFSI), solvents (*e.g.*, DME, DOL) and additives, *e.g.*, fluoroethylene carbonate (FEC) and LiNO_3_ that tackle inhomogeneous Li electrodeposition in Li metal cells. Here, we used FEC to prepare an optimal electrolyte as compared to the standard LP30. DOL : DME electrolytes are studied here too, as they are used in Li sulphur batteries. Specifically, 1 M LiTFSI in DOL and DME (referred to as DOL : DME) is usually used with the additive LiNO_3_ since it promotes a more stable SEI and plating. We intentionally used this electrolyte without the additive so as to promote the poor cell behaviour we needed to allow for comparison with the more optimised electrolytes.

## Results

### Typical voltage profiles for symmetric Li cells

**Fig. 2:**
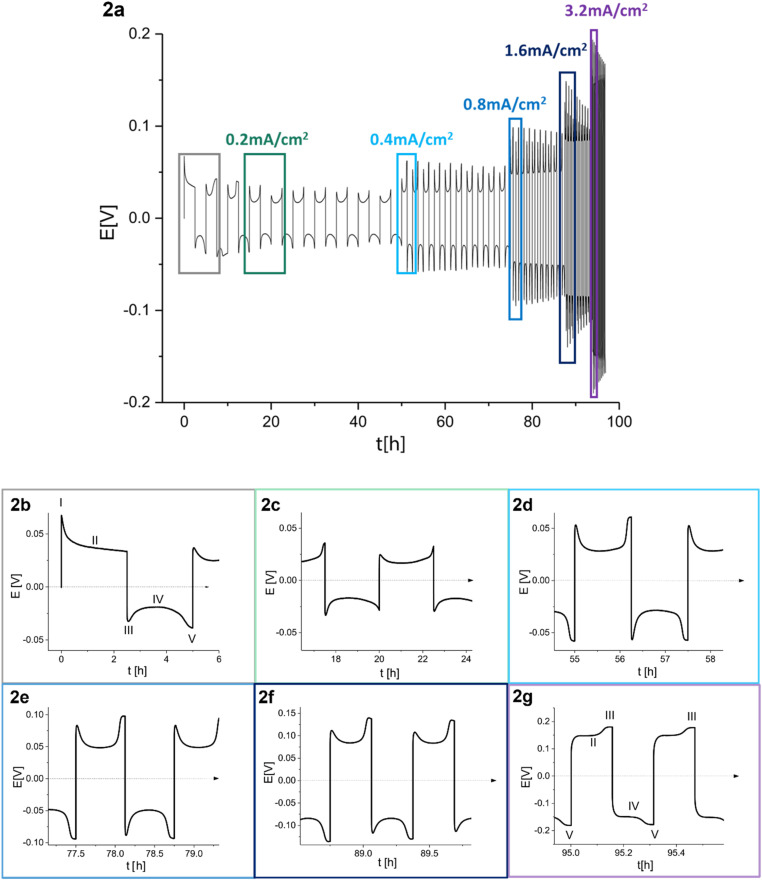
(a) Voltage trace (*E vs. t*) of successive galvanostatic cycling of a total of 1 mA h cm^−2^ of Li at increasing currents of 0.2, 0.4, 0.8, 1.6 and 3.2 mA cm^−2^ in lithium symmetric cell with 1 M LiPF_6_ in EC : DMC (LP30). (b)–(g) Magnification of selected voltage traces (*E vs. t*) of successive galvanostatic cycling of a total of 1 mA h cm^−2^ of Li at increasing currents of (b) 0.2, (c) 0.2, (d) 0.4, (e) 0.8, (f) 1.6 and (g) 3.2 mA cm^−2^ in lithium symmetric cell with 1 M LiPF_6_ in EC : DMC. For 0.2 mA cm^−2^ both the first (b) and the fourth (c) cycles are presented. Characteristic peaks and processes are labelled, following the assignments made by Dasgupta *et al.*^1,2^

The Li metal plating dynamics and the corresponding short circuit formation were studied in symmetric Li coin cells. Galvanostatic plating and stripping cycles were conducted at current densities 0.2–3.2 mA cm^−2^ in 1 M LiPF_6_ EC : DMC 1 : 1 (referred to as LP30) (Fig. 2). The study of the voltage traces follows the methodology introduced by Dasgupta *et al.*^1,2^ The amount of cycled Li was kept constant while increasing currents were applied for decreasing periods. The peaking behaviour is consistent at 0.2 and 0.4 mA cm^−2^ (Fig. 2b–d), while the sharpness of the peaks decreases between 0.8 and 1.6 mA cm^−2^ (Fig. 2e and f). The voltage “arches” up, *i.e.*, increases noticeably at 3.2 mA cm^−2^, and sometimes a double plateau is seen (Fig. 2g).

During the first half-cycle (Fig. 2a, magnified in Fig. 2b), pitting occurs on the lithium anode (the electrode where the electrooxidation process occurs) due to the non-homogeneous electro-dissolution of lithium, while metal nucleation occurs on the cathode (the electrode where electrodeposition occurs). An additional energy barrier introduced by the nucleation results in the first local voltage maxima (peak I) with additional overpotential *η*_ac_. Additional lithium growth takes place on the nucleates on the cathode (rather than forming additional nucleates), resulting in a drop in the cell overpotential, which is followed by a voltage plateau (II). After switching the current polarity, electrooxidation occurs from the Li microstructures (which can be mossy or dendritic depending on a variety of factors including the electrolyte, the current density and temperature). A maximal voltage is observed due to lithium nucleation on the anode (peak III). As the electrodeposition continues, voltage plateau IV is seen, which is followed by a second peak at the end of the half-cycle (peak V). Peak V results from the pitting of the bulk metal, which starts after the consumption of all the microstructures on the anode that were formed during the previous half-cycle. This peak occurs before the end of the cycle due to the low efficiency of lithium plating (and stripping) caused mainly by the constant breakdown and repair of the SEI as the volume of the deposited metal continuously changes.

The voltage traces evolve during prolonged cycling. To illustrate, the voltage trace of a Li symmetric cell, cycled for 55 and 95 hours, is depicted in Fig. 2d and g, respectively. The flat shape of peak III (following the labelling in Fig. 2b) was attributed by Dasgupta *et al.* mainly to dendrites that form in the pits. The flat shape of peak III, followed by an overpotential increase during stages II and IV, results in an arch shape of the voltage trace (referred to as arching; note that somewhat confusing terminology does not indicate an arcing process). Dasgupta *et al.* showed that the transition from “peaking” to “arching” behaviour is attributed to issues that develop during prolonged cycling by accumulating the dead lithium, which introduces a tortuous pathway for ion transport.^1,2^

**Fig. 3:**
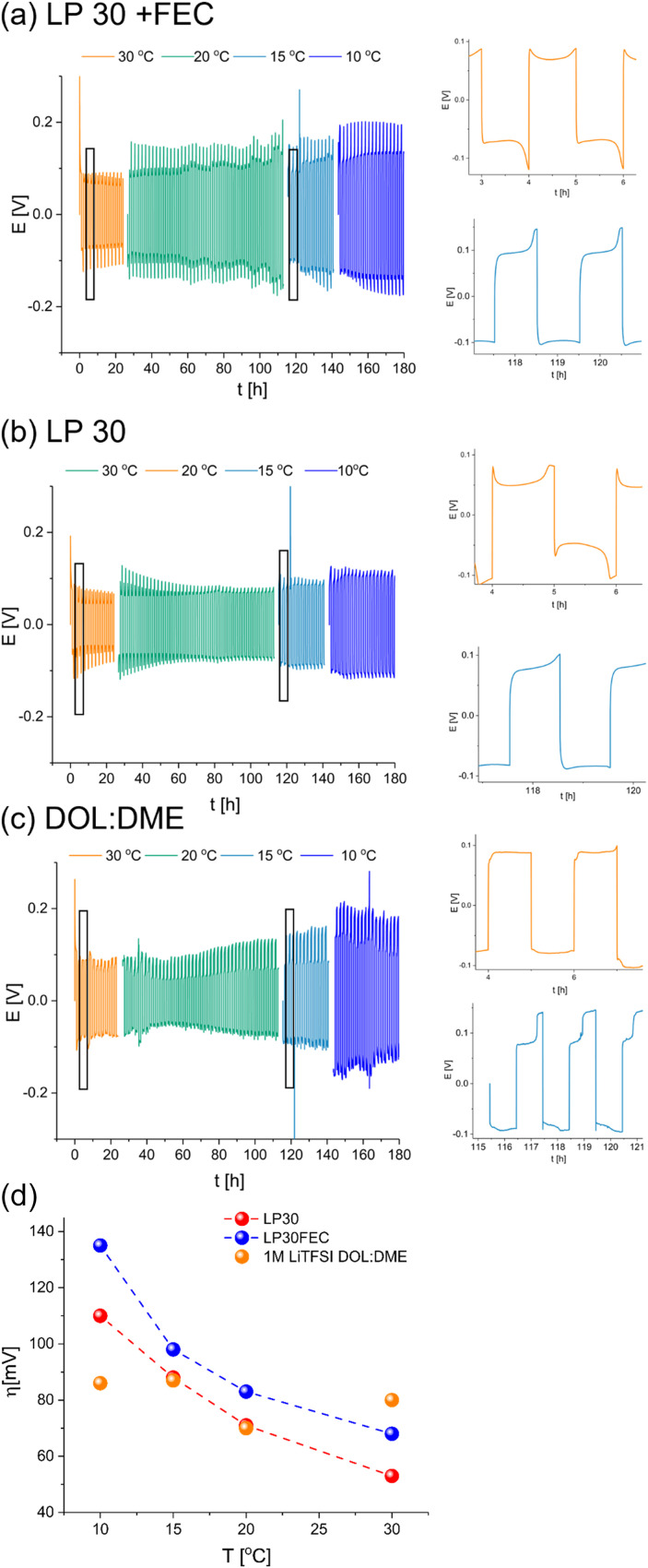
Voltage traces of Li symmetric cells with Celgard–GF–Celgard separators at 30 °C (orange), 20 °C (green), 15 °C (turquoise) and 10 °C (blue) in (a) LP30 + FEC, (b) LP30 and (c) DOL : DME at the top, middle and bottom, respectively. The cycles recorded at 15 °C and 30 °C were magnified. (d) Overpotential evolution with temperature in Li symmetric cells with Celgard–GF–Celgard separators (used for preventing a hard short circuit) and LP30 (red), LP30 90% and FEC 10% (blue) and 1 M LiTFSI DOL : DME 1 : 1 (orange) electrolytes.

Li symmetric cells based on either LP30, LP30 with 10% v/v FEC (referred to as LP30 + FEC) and DOL : DME with a tri-layered Celgard/GF/Celgard separator were cycled at 1 mA cm^−2^ in symmetric cells at 10–30 °C (Fig. 3). The Celgard–GF–Celgard separators layer was used to prevent a hard short circuit.

To explore the evolution of the charge transport in the cell during Li plating, these cells were cycled in different temperatures and relatively high current density (1 mA h cm^−2^). Since the SEI regenerated every cycle, its thickening and degradation is neglectable, therefore the overpotential change with temperature reflects the nature of the rate determining charge transport step. The cycling in carbonate-based electrolytes resulted in the typical double-peaking voltage traces at 30 °C and then arching at low temperature (15–10 °C) after prolonged cycling (Fig. 3). In contrast, cycling of Li in DOL : DME electrolyte-based cells resulted in a rectangular-shaped voltage trace at 30 °C (although minor peaks are visible), which transformed into a trace composed of arching voltage traces with two well-separated plateaus on the cathode (the Li electrode on which plating took place at the first cycle) and a characteristic needle-like peak V followed by arching on the anode (the Li electrode on which stripping took place at the first cycle). The needle-like shape of the voltage traces in DOL : DME (Fig. 3c) was replaced by a rectangular-shaped trace when a single GF separator was used (Fig. S1c,† bottom).

An Arrhenius plot of the overpotential measured during stage II of the cycling of these cells was made to study the effect of temperature on the overpotential traces. The cell was left to equilibrate at rest for two hours after each temperature change and then to complete an entire galvanostatic cycle. Then, the overpotential measured during stage II was used to construct the Arrhenius plot. For the galvanostatic cycling at 10 °C, the overpotential of stage II was taken from the last cycle to encounter the rapid degradation typical for this temperature.

While the overpotential for plating in LP30 and LP30 + FEC electrolytes correlates negatively with temperature, the overpotential of lithium plating in DOL : DME is approximately constant in the tested temperature range. This is in contrast to the expected trend for a system where the charge transport is dominated by ion transference, indicating the presence of a significant electron transport component (Fig. 3d).

In another set of experiments, a single GF separator was used instead of a tri-layered Celgard/GF/Celgard. In this case, the separator is not likely to prevent short-circuiting; the cycling resulted in rectangular-shaped voltage traces for the DOL : DME cells (Fig. S1c†).

The cycling of the cells with the FEC additive, which improves charge transport in the SEI and plating,^5^ results in more pronounced peaking compared to cells with LP30, in which the peaking shape of the voltage traces transformed into arching during cycling (Fig. 2). The Arrhenius plot for the carbonate electrolyte-based cells showed similar trends, while the overpotential of the DOL : DME cells correlates positively with temperature (Fig. S1b†).

### Prolonged plating and galvanostatic impedance spectroscopy (GEIS) studies

**Fig. 4:**
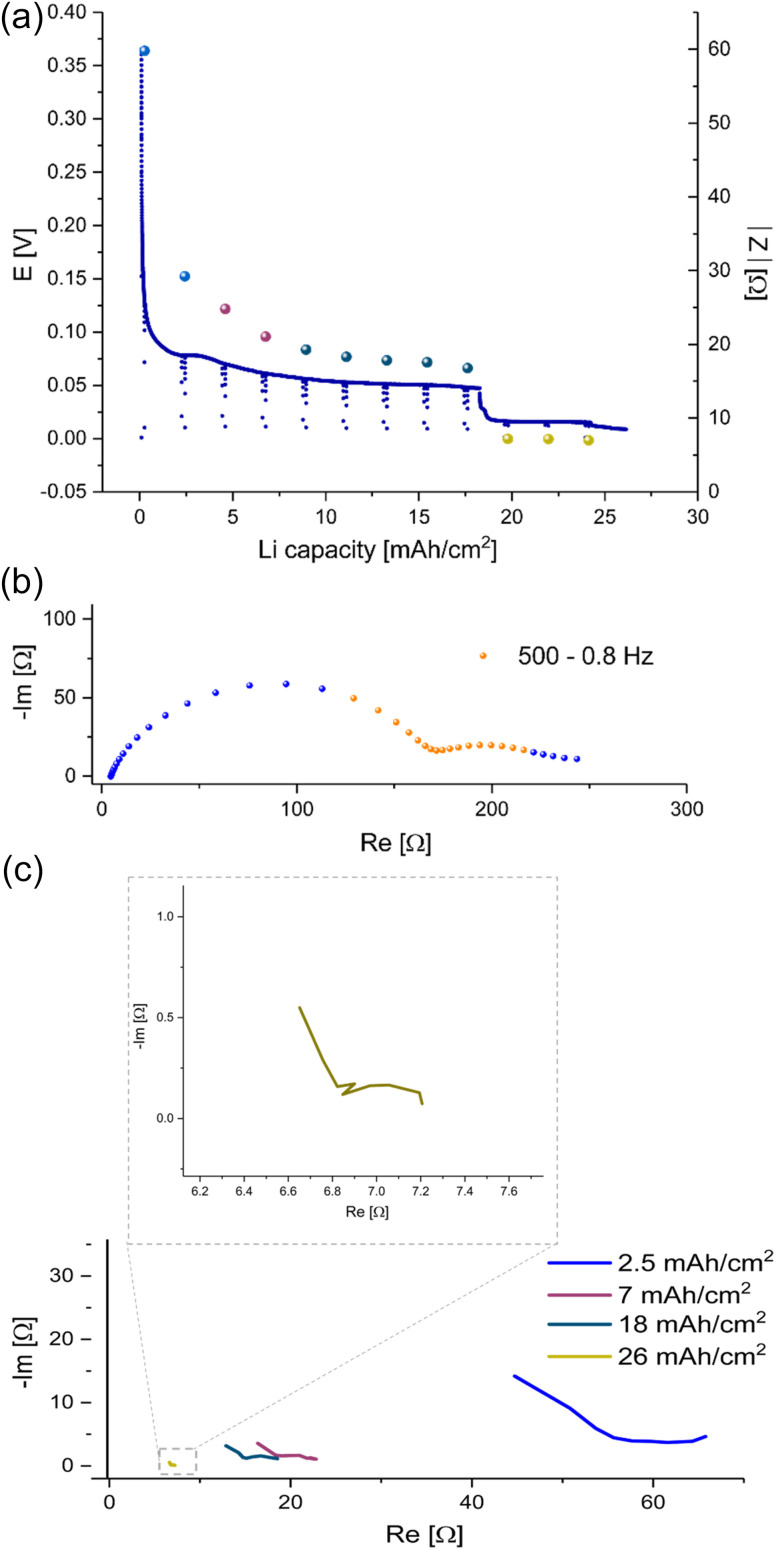
(a) The voltage trace (*E*–*t*) of galvanostatic plating a total of 26 mA h cm^−2^ Li at 1 mA cm^−2^ in Li symmetric cell with LP30 electrolyte and GF separator at RT. EIS module values (spheres) were measured with GEIS at 9 Hz with 100 μA amplitude at 500–0.8 Hz. (b) A Nyquist plot of the PEIS spectrum of a symmetric lithium cell, LP30 electrolyte with GF separator measured after native SEI formation and before Li plating with 10 mV amplitude at RT. The frequency range used in the following GEIS measurements is indicated in orange. (c) Nyquist plots measured with GEIS, 100 μA amplitude at RT during Li plating in a symmetric cell, LP30 electrolyte RT with GF separator. Magnification of the plot measured after plating 26 mA h cm^−2^ Li.

To determine the effect of prolonged plating on charge transport, galvanostatic impedance spectroscopy (GEIS) was used. GEIS was chosen to probe the impedance of the cells, with minimal effect on the cell (*i.e.* plating and SEI formation). The impedance intensity around 9 Hz was plotted alongside the *E*–*t* curve (Fig. 4a). This approach was validated by comparing the trend of the impedance intensity in the medium frequency range (Fig. S2†), where the measurements at 9 Hz were shown to follow similar trends to the ones recorded at the 500–8 Hz range.

26 mA h cm^−2^ of Li was plated at 1 mA cm^−2^ and the impedance was measured using the GEIS method every 2.5 hours. The impedance at 9 Hz was plotted with the voltage trace in Fig. 4a, and the corresponding Nyquist plots are depicted in Fig. 4b and c. During the first eight hours (plating approximately 8 mA h cm^−2^ Li), the impedance of the cells decreased linearly. After ten hours of plating, the impedance decrease stopped, and a voltage plateau was seen. After 26 hours of plating (approximately 26 mA h cm^−2^ Li), the impedance abruptly dropped below 10 Ω (approximately 0.2 mS cm^−2^) concurrently with a drop in overpotential of the cell.

Prolonged unidirectional plating of lithium (Fig. 4a) resulted in an initial impedance drop, followed by a linear decrease, a plateau and then an abrupt decline to very low impedance values. At the same time, the corresponding Nyquist plots indicate changes in the charge transfer after eight hours (Fig. 4c). Hence, we assigned the three stages of plating to metal nucleation, metal surface area growth, and soft shorts formation, respectively. The EIS spectra (Fig. 4c and S3 in the ESI†) measured after plating 7 mA h cm^−2^ differ from those measured during the first 2.5 mA h cm^−2^. While the dominant semi-circle in the spectrum recorded at the beginning of the plating has a typical capacitance for an SEI.^9^ However, the spectrum measured after plating of 7 mA h cm^−2^ consists of two semi-circles. One has a typical capacitance for an SEI, and the other has a capacitance typical for a longer diffusion length, suggesting a mixed charge transport (see ESI part 2 Fig. S3 and part 5, Fig. S12 and S13†). We attribute this charge transport change to the unique response of a soft shorted cell.

**Fig. 5:**
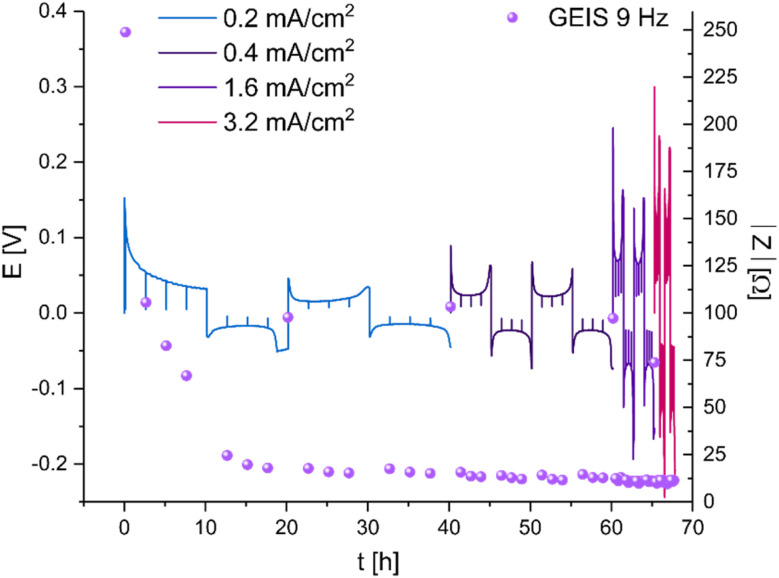
Voltage traces (*E*–*t*) of galvanostatic plating of 1 mA h cm^−2^ Li at increasing current densities between 0.2 and 3.2 mA cm^−2^ in a Li symmetric cell, LP30 electrolyte and GF separator at RT. EIS intensity (purple spheres) measured with GEIS at 9 Hz with 100 μA amplitude.

To explore these phenomena under more realistic conditions, a new series of symmetric cells were cycled at current densities between 0.2 and 3.2 mA cm^−2^. The amount of cycled lithium was kept constant (1 mA h cm^−2^) (Fig. 5). While the overpotential increases linearly with the current, the charge transfer resistance, measured *via* GEIS, decreases abruptly ten times during the first two cycles and remains around 10 Ω (approximately 0.2 mS cm^−2^). From this point, the impedance of the cell equals approximately the ohmic resistance, calculated by dividing the measured overpotential by the applied current. Replacing the GF separator with a Celgard separator resulted in similar trends (Fig. S4†). Notably, the initial impedance of the non-cycled LP30–Celgard cell is significantly higher. When the Celgard separator was used, a more pronounced peaking behaviour of the voltage trace was observed. The impedance drops sharply during the first ten hours of cycling; then, it remains stable; however, higher values are measured consistently when the polarity of the current is reversed (*i.e.* when the freshly plated microstructures are being oxidised).

A similar trend was demonstrated while plating at 3.2 mA cm^−2^ at 10 °C, conditions under which dendritic plating and short circuits are more likely (Fig. S5–S7†). While impedance recovery was demonstrated in all the LP30-based cells, in the DOL : DME-based cells, the impedance remained at very low values throughout the cycling, also when the current was reversed. The voltage traces in the LP30 and LP30 + FEC cells have a peaking shape, the galvanostatic cycling of Li in DOL : DME results in typical arching voltage traces.

### *Operando* NMR experiments under GEIS cycling

**Fig. 6:**
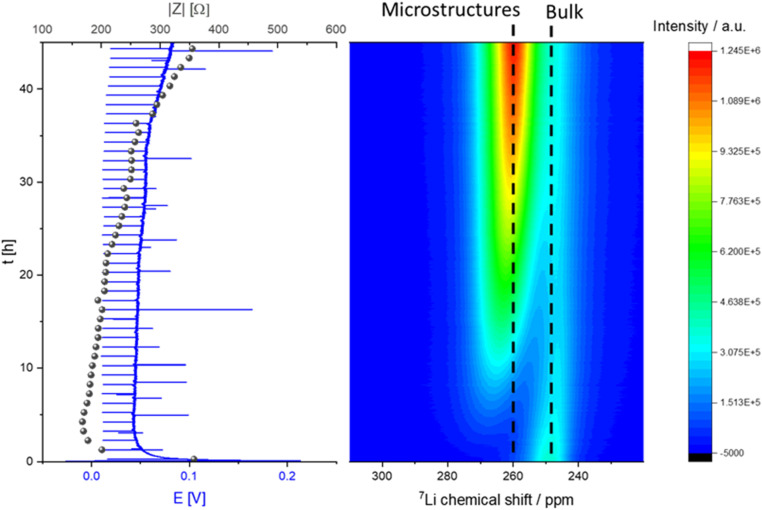
*Operando* NMR spectra of unidirectional Li deposition in an NMR *in situ* cylindrical symmetrical Li cell with LP30 electrolyte (right) with corresponding voltage profile and impedance (GEIS) intensity measured at 9 Hz (left) during galvanostatic plating with a current density of 0.5 mA cm^−2^.

NMR studies were performed for cycling for prolonged times (up to 45 hours) to investigate the metal deposition and short-circuiting in symmetric Li cells. Here, *in situ* GEIS measurements were recorded simultaneously for the first time, allowing us to decipher in more detail changes in the NMR spectra. In the ^7^Li *operando* NMR spectra two different signals are visible, the peak at around 0 ppm can be assigned to the diamagnetic ^7^Li nuclei which correspond to the electrolyte and the SEI (Fig. 6).^10^ The signal at *ca.* 245 ppm could be assigned to the pristine Li metal of the two electrodes in the cell.

The micro-structured and bulk metal can be distinguished using the chemical shifts caused by the bulk magnetic susceptibility (BMS) effect. Aligning the cell perpendicular to the static magnetic *B*_0_ field brings the BMS into play. This signal is described as “bulk metal” signal in the following discussion of the results. The large shift in the metal peak is caused by the Knight shift, which is characteristic for metallic samples and a measure of the density of states at the Fermi level.^11^ During constant galvanostatic plating an additional peak appears with a chemical shift of *ca.* 260 ppm. This new signal is constantly growing during the experiment and is indicative for the formation of microstructures on the surface of the metal electrodes (Fig. 6). Furthermore, the skin depth effect (see Experimental – *Operando* NMR) must be considered when integrating the metal peaks in the spectra.

**Fig. 7:**
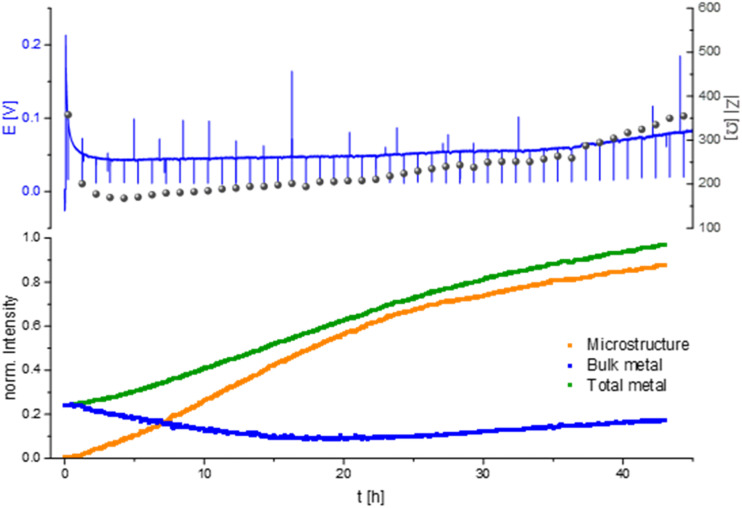
*Operando* ^7^Li NMR and impedance (GEIS) measured at 8 Hz. Intensities measured during unidirectional Li plating at 0.5 mA cm^−2^ in an NMR *in situ* cylindrical symmetrical Li cell with LP30 electrolyte.

A characteristic voltage and impedance traces (Fig. 7) is seen when measuring *operando* NMR plating Li with a current density of 0.5 mA cm^−2^ in an LP30 electrolyte. The overpotential drops at the beginning and remains stable until increasing at later stages (approximately after 30 hours) due to the formation of more resistive SEI. The impedance follows the trend of the overpotential profile until reaching the maximum impedance of approximately 300 Ω at the end of the experiment. The GEIS Nyquist plots (Fig. S8†) comprise a semi-circle at the medium frequency with a capacitance of approximately 10^−6^ F (see semi-circle fits in Fig. S12 and S13†). The intensity of the impedance follows the overpotential trend decrease during the first hours of plating and then a constant increase, while the shape of the Nyquist plots remains unchanged throughout the experiment.

When quantifying the metal signals, it is clearly visible that the total metal signal, which is indicative for the surface area, is increasing nearly linearly during the experiment. In addition, a linear increase in the microstructural lithium metal peak is detected until 22 hours of the experiment. After 22 hours the slope of the microstructure growth becomes more moderate. The bulk metal signal, however, shows a different trend. When starting the plating the bulk metal signal decreases until the 22nd hour of plating and afterwards its intensity starts to increase slightly until the end of the experiment (Fig. 6 and 7).

**Fig. 8:**
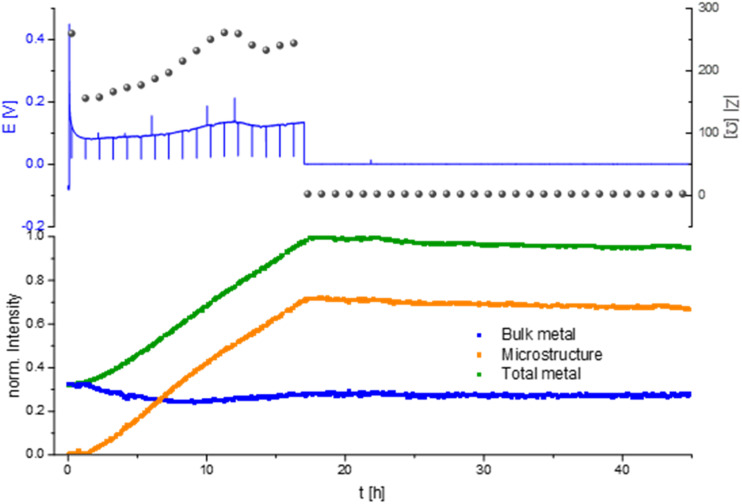
*Operando* ^7^Li NMR and impedance (GEIS) measured at 8 Hz intensities measured during unidirectional Li plating at 1 mA cm^−2^ in an NMR *in situ* cylindrical symmetrical Li cell with LP30 electrolyte.

Measuring the Li plating at higher current density, 1 mA cm^−2^ in an LP30 electrolyte shows a characteristic voltage and impedance profile (drop at the beginning and afterwards a constant increase) until the 17th hour of the experiment (Fig. 8). During the first 17 hours, the GEIS Nyquist plots (Fig. S9†) comprise two semi-circles with a similar diameter. The intensity of the impedance follows the overpotential trend, while the shape of the Nyquist plots remains unchanged throughout the experiment. During this time the intensity of the total metal signal and the microstructure peak increase linearly, consistent with previous measurements.

After 17 hours the voltage and the impedance drop to 0, clearly indicating a hard short circuit of the cell. The GEIS Nyquist plot loses its semi-circles and shows a typical shape for hard short. When the overpotential drops the intensity of the metal peaks remained nearly constant with a slight decrease in the total metal and microstructure intensity of 5% until the end of the experiment, which we attribute to corrosion of Li metal.^12^

**Fig. 9:**
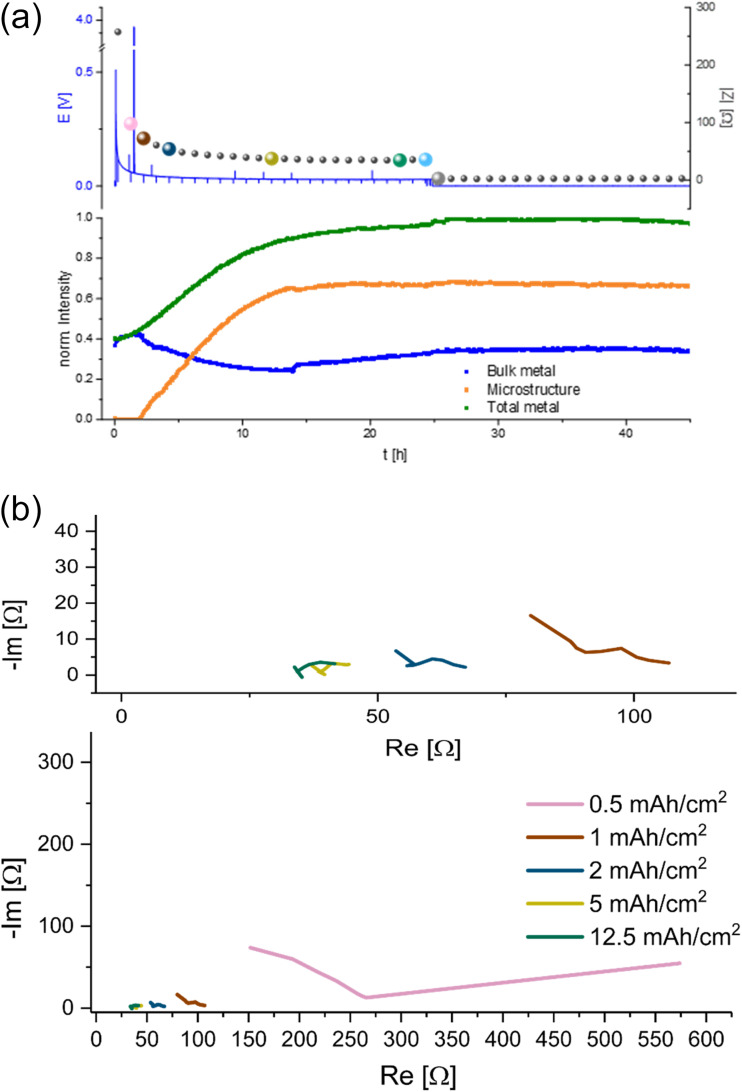
(a) *Operando*^7^Li NMR and impedance (GEIS) measured at 9 Hz. Intensities measured during unidirectional Li plating at 1 mA cm^−2^ in an NMR *in situ* cylindrical symmetrical Li cell with LP30 + 10% FEC electrolyte. (b) GEIS Nyquist plots measured with 100 μA amplitude during Li plating at 1 mA cm^−2^ in an NMR *in situ* cylindrical symmetric cell, using LP30 + FEC electrolyte with GF separator at RT. Magnification of the plot at 5–15 mA h cm^−2^.

A different behaviour of the plating is detected in the electrolyte with the additive FEC (Fig. 9a). During the NMR experiment the cell showed a typical smooth voltage profile during plating and relatively low, decreasing overpotential and impedance. After approximately 25 hours of unidirectional plating, the overpotential and impedance dropped to 0 and did not change until the end of the experiment. Quantifying the metal signals of this plating experiment shows a nearly linear increase in the microstructure and the total metal peak until 10 hours of the experiment. After 10 hours the increase becomes more moderate, until it stops after 15 hours of plating and stays nearly constant with a slight decrease of 2% until the end of the experiment. The bulk metal signal decreases in the first 15 hours, increases in the following nine hours and then remains constant until the end of the experiment.

The shape of the Nyquist plots of the GEIS measured during plating (Fig. 9b) transforms during the unidirectional plating. A typical double semi-circle-based shape, measured after 0.5 and 1 mA h cm^−2^. After plating 2 mA h cm^−2^ Li, the maximum frequency of the SEI semi-circle shifted towards higher frequencies. The second semi-circle has remained at low frequencies (from approximately 200 Hz to 5 Hz) and capacitance of mF (see Fig. S13 in the ESI†).

## Discussion

Lithium metal symmetric cells are widely used to study metal anodes, electrolytes and interfaces. Typically symmetric cells are characterised *via* galvanostatic cycling at a constant current density, followed by an analysis of the voltage profile. This study explored whether soft-shorts can be detected during Li metal plating and whether rectangular-shaped voltage traces (Fig. 3c and S1a bottom†) indicate effective Li metal plating.

Here we showed that even the initial voltage traces of galvanostatic Li plating, GEIS intensity and Nyquist plots of Li symmetric cells could indicate the tendency for soft shorts. As has been shown in numerous studies, LP30 + FEC electrolyte is more effective in Li plating; seen by the voltage traces in Fig. 3a, S1a and S5.† It results in initial double-peaking voltage traces that evolve to arching after prolonged cycling in variable temperatures (Fig. 3a and b and S1a top and middle†). In contrast, using the DOL : DME electrolyte under the same conditions gives rise to either rectangular-shaped voltage traces or characteristic arching with needle-like peaks (Fig. 3c and S1a†). Since needle-like and rectangular voltage traces appear after cycling under very similar conditions (Fig. 3a and b and S1a top and middle†), we assign them to different stages in the development of soft shorts. Moreover, we suggest that arching, which was previously assigned to dead Li formation, could be used as an indication of upcoming soft shorts formation. We aim to validate this hypothesis in future research.

The trend in overpotential with the temperature reflects the nature of charge transport (Fig. 3d and S1b†) *i.e.*, when ion migration dominates charge transport, the overpotential will correlate negatively with temperature as seen for the carbonate-based electrolytes (Fig. 3d and S1b†). In contrast, a typically poor-performing electrolyte (1 M LiTFSI DOL : DME, without additives) gave rise to an opposite trend, which we attributed to electronic charge transport, typical for a soft short circuit.

We demonstrated that the impedance of lithium symmetric cells decreases during unidirectional plating and galvanostatic cycling. The decrease in the impedance can be attributed to the increased surface area arising from Li deposits as shown by *operando* NMR. However, an abrupt drop in the impedance to very low values during prolonged unidirectional plating of lithium (Fig. 4a, 6, 8, 9a, S10 and S11†) relates to soft shorts formation. This is shown in Fig. 4, where after seven hours of plating at 1 mA cm^−2^, in addition to the impedance intensity drop, the semi-circle typically associated with the charge transport shifted to a higher frequency, while clearer low frequency semi-circle with a capacitance in the order of mF appeared. Both are previously associated with a longer charge transport path and a more porous SEI.^13^ We attribute these transitions to a change in the nature of the charge transfer as a result of the soft shorts, as corroborated by the NMR data in Fig. 9, S10 and S11,† where both the GEIS and NMR spectra are indicative of soft shorts significantly earlier than the permanent short formation. Notably, the appearance of these indicators is significantly earlier for plating at 1 mA cm^−2^ (Fig. 9) compared to 0.5 mA cm^−2^ (Fig. S10 and S11†); however, the trend in the GEIS is very similar in the three experiments compared.

The dynamics of soft shorts were demonstrated in realistic cycling conditions using a polarisation procedure typically used to determine the critical current density. While the overpotential increases linearly with the current, the impedance that represents the charge transfer resistance decreases abruptly ten times during the first two cycles and remains around 5 Ω cm^−2^ throughout the cycling, except for a few recovery reoccurring events (Fig. 5). The transient recovery events, recorded for GF and Celgard-based cells (Fig. 5 and S4†), represent the breakdown of the electron pathway, typical for the soft short. We assigned this combination of abrupt impedance drop and periodic recovery to soft shorts formation and the current density at which the impedance stops to recover as an alternative definition to the critical current density, which predicts potential cell failure.

The constant increase in the total metal peak in the NMR experiment corresponds with the formation of a high surface area during the whole plating experiment. It can be correlated with the trends in the microstructure signal and soft shorts formation. The linear increase in the peak with a higher chemical shift of 260 ppm during the first 22 hours of plating in LP30 (Fig. 6 and 7) shows a constant building of microstructure on the surface of the lithium metal electrode. The bulk metal peak decreases in the first 22 hours of plating. An attenuation of the radiofrequency (*B*_1_) field can explain this phenomenon by forming dense microstructure on the electrode surface because the radiofrequency penetration into the bulk metal is less than that for the initial Li electrode.^14,15^ After 22 hours, further plating occurs on the electrode. In addition to forming more surface area, the gaps between the lithium micro-structures are filled. Then the bulk metal intensity is slightly increasing again due to the formation of denser surface structures. We demonstrated this phenomenon in our previous work.^5^

Prolonged Li plating at higher current density (1 mA cm^−2^) in LP30 resulted in a hard short circuit after 17 hours of plating (Fig. 8). The micro-structured lithium metal and the total metal intensity stopped increasing due to metal plating termination and the formation of a short circuit. After short-circuiting, the signal intensity of the total metal (indicative of the surface area) and the micro-structured lithium metal decrease, which the corrosion of the microstructure can explain with an ongoing decrease of the surface area.

Unidirectional plating at 1 mA cm^−2^ in a Li symmetric cell based on LP30 + FEC electrolyte resulted in a different behaviour, consistent with previous studies of the FEC additive (Fig. 9). Lower plating overpotential and a smooth plating curve likely result from faster Li-ion transport and faster healing of the SEI in FEC-containing cells. Although the voltage profile indicates a continuous metal plating after 15 hours, the termination of microstructure formation indicates that the plating stopped. Considering the constant low impedance intensity and the significant changes in the Nyquist plot, we attributed this unique behaviour to the beginning of a soft short formation. Moreover, we found that this behaviour is typical for carbonate electrolytes by demonstrating similar trends in a coin cell (Fig. 4). Hence, the NMR indicates the termination of plating while the GEIS the formation of the short (see further discussion in ESI part 6 Fig. S14 and S15†). The slight decrease in the total metal intensity after about 25 hours, during the soft short circuit state, can be attributed to the corrosion of the Li microstructure.

## Conclusions

We showed that soft short circuits are common in lithium cycled at realistic conditions; however, they are typically overlooked. The familiar rectangular-shaped voltage traces, typical for symmetric metal cells, were proven as misleading since they are usually assigned to effective Li metal cycling, resulting from soft shorts and potentially explaining the significant performance gap between symmetric cells and realistic metal batteries.

Here, soft short circuits formed during prolonged unidirectional Li plating were detected with coupled EIS and NMR, where three stages of Li plating: nucleation, surface area growth and soft shorts formation were distinguished.

We have shown that exceptionally low overpotentials and arching or rectangular shaped voltage traces are often a result of evolving soft shorts, demonstrated that even the initial voltage traces reflect the tendency of the cell to short. Combining galvanostatic cycling with *in situ* GEIS measurements can be used to predict degradation at a very early stage. We suggest that both the unique arching shape of the voltage trace and the sharp impedance drop are typical for already soft-shorted cells (or cells about to become shorted). Electrolytes that typically encourage a less dendritic Li morphology and have a lower tendency for Li corrosion (as we showed in our previous study^12^) have a lesser tendency for soft shorts formation.

The existence of reversible soft shorts, measured during a symmetric cell polarisation experiment, suggest that the critical current density should be redefined to reflect the current density at which the soft shorts are not recoverable anymore. Moreover, using coupled *operando* NMR and GEIS for the first time, we demonstrated that soft shorts are predictive towards forming hard shorts, demonstrating the strength of *operando* NMR as a research tool and enabling a fundamental understanding of Li plating. Even more importantly, we showed that medium-frequency GEIS (a readily available and relatively low-cost technique) could be used to predict upcoming battery catastrophic failure.

The impact of this work is beyond more effective symmetric cell methodology. The insights are expected to alter the research of metal battery degradation, enable faster realisation of high energy anode-free batteries and contribute to the fundamental understanding of metal plating.

## Experimental

### Cell assembly

Cell assembly and air-sensitive material handling were done in an argon glovebox (MBraun, O_2_, H_2_O < 1 ppm). The electrolytes used were the following: 1 M LiPF_6_ in ethylene carbonate and dimethyl carbonate (EC : DMC 1 : 1 volume ratio, Sigma-Aldrich, battery grade), termed LP30 in this study. LP30 + FEC was prepared by mixing LP30 with fluoroethylene carbonate additive in a 9 : 1 volume ratio (FEC, Sigma-Aldrich, 99%). The electrolyte referred to as DOL : DME was prepared using 1 M lithium bis(trifluoromethane sulphonyl)imide (LiTFSI, Sigma-Aldrich, 99.95%) in 1,3-dioxolane (Acros Organics, anhydrous, 99.8%) and 1,2-dimethoxyethane (Merck, anhydrous, 99.9%) (DOL : DME in 1 : 1 volume ratio). The LiTFSI salt was dried for 20 h at 120 °C under a vacuum before use.

Lithium metal disks (15.6 mm × 0.25 mm thick) were purchased from PI-KEM, opened, and stored in an argon glovebox, used as received. Stainless steel 304 coin cell parts (Cambridge Energy) were sonicated in ethanol and dried at 60 °C overnight prior to cells assembly. Glass fibre (Whatman GF/A) and Celgard (polyethylene–polypropylene layered) separators were used after being dried in vacuum at 100 and 40 °C, respectively.

A capsule cell (NMR Service) made out of PEEK (polyether ether ketone) was used for all *in situ* NMR experiments and has been described before.^16,17^ Working electrodes consisted of a Li metal foil. The amount of electrolyte added to each cell was 100–150 μL.

Coin cells consist of two bases, a spring, two 0.49 mm stainless steel disks, two Li electrodes, and a Celgard or a glass fibre (Whatman GF/B) or ADVANTEC GC-50 separators with 75–100 μL electrolyte, respectively.

### Electrochemistry

BioLogic MPG2 cyclers were used for galvanostatic cycling. A BioLogic VSP-300 cycler was used for impedance spectroscopy, and a BioLogic VSP cycler was used during *operando* NMR experiments. EC-Lab software (V11.32) was used for data collection and processing.

For symmetrical Li cells, the constant current was applied in either a single direction or cycling at 0.2–3.2 mA cm^−2^ current density while maintaining a constant amount of electrodeposited Li.

### Galvanostatic cycling

Galvanostatic cycling of Li symmetric cells was done at constant 1 mA h cm^−2^ Li at 0.2, 0.4, 0.8, 1.6 and 3.2 mA cm^−2^ in lithium symmetric cells. A constant capacity (the amount of Li plated and stripped at each half-cycle) was maintained constant throughout the cycling and polarisation (*i.e.*, critical current density tests).

### Variable temperature galvanostatic cycling

Li symmetric cells were cycled for ten 2 hour cycles at 1 mA cm^−2^ at 30 °C, 20 °C, and 10 °C, then cycled at 10 °C until the cells failed. The two hours of rest were followed by each temperature change to allow the cells to equilibrate.

### Galvanostatic unidirectional plating

Galvanostatic Li plating in symmetric cells was done at 0.2, 0.4, 0.8, 1.6 and 3.2 mA cm^−2^ current density. A total Li capacity of 20–40 mA h cm^−2^ was plated to measure the response of the symmetric cells system at extreme conditions, which are not likely to be used in a practical metal battery (usually, the areal capacity is up to 10 mA h cm^−2^).

### Impedance

Electrochemical impedance spectroscopy (EIS) experiments are either galvanostatic (GEIS) or potentiostatic (PEIS). The SEI resistance governs the impedance of Li metal symmetric cells. In a new cell, the native SEI formed on the Li electrodes is relatively resistive (a total impedance intensity (|*Z*|) of 300–2000 Ω cm^−2^ measured in coin cells). However, the resistance typically abruptly drops with the application of current. Hence PEIS, using a constant voltage amplitude (typically 10 mV), will result in a minimum signal-to-noise ratio. Measurements were taken at frequencies between 0.1 Hz and 1 MHz, six measurements were taken per decade with a logarithmic spacing, and each was measured twice.

However, in cycled symmetric Li cells, the SEI is freshly formed and relatively thin (*i.e.*, low resistance, typically a total impedance intensity (|*Z*|) of approximately 50 Ω cm^−2^ measured in coin cells), hence the application of 10 mV voltage amplitude will result in a significant current and potentially modify the conditions in the cell. Thus, GEIS with an amplitude of 100 μA was used when measuring *in situ* during galvanostatic cycling. Measurements were taken at frequencies between 500 and 0.8 Hz, three measurements per decade with a logarithmic spacing, and each was measured once. The narrower range, fewer measurements, shorter measurement time and lack of repeats minimised the effect on the symmetric cell.

### Galvanostatic cycling with *in situ* GEIS

To probe the impedance while cycling, EIS measurements were implemented into the galvanostatic cycling profile. A PEIS measurement (10 mV amplitude, 1 MHz to 0.1 Hz) was done after two hours of storage and prior the formation. Then, the SEI was formed (galvanostatic for 10 minutes) and then the first GEIS measurement was taken, followed by either GEIS probing (100 μA amplitude, 500–0.8 Hz) every 2–2.5 hours during unidirectional plating or cycling. The voltage spikes, apparent in all the galvanostatic measurements in this work (Fig. 4a, 5, 6a, 7a and 8a), result from the effect of the GEIS measurements.

When GEIS was combine with galvanostatic cycling, the GEIS measurements were taken after the current direction was switched (stages III, V Fig. 2a) and at the middle of the plating (stages II, IV Fig. 2a).

### *Operando* NMR coupled with galvanostatic plating and GEIS

For the *in situ* NMR cell setup in this study, all the cells operate with the applied current densities 0.5–1 mA cm^−2^. The calculated limiting current density^2^ is ∼7 mA cm^−2^. Thus, the galvanostatic experiments performed in this work are at the low current density regime.^18^

### *Operando* NMR

The *operando* NMR experiments were conducted on a Bruker Avance 300 MHz spectrometer (the Larmor frequency for ^7^Li being 116.6 MHz) using a solenoidal Ag-coated Cu coil. The spectra were recorded using an *in situ* automatic-tuning-and-matching probe (ATM VT X *in situ* WB NMR probe, NMR Service) that allows for an automatic recalibration of the NMR rf-circuit during an *in situ* electrochemistry experiment. The retuning of the rf-circuit becomes essential in order to quantify the NMR signals when the sample conditions are changing during the electrochemistry.^19^ The probe has highly shielded wire connections to the electrochemistry with low-pass filters (5 MHz) attached to the probe, minimizing the interferences between the NMR and the electrochemistry circuit, as described in a previous publication.^19^

Overall, the *in situ* setup allows for highly reproducible NMR measurements.^19^ Single-pulse experiments were used to collect the NMR data, with a recycle delay of 1 s (>5 × T1) and 256 transients recorded. This resulted in an experimental time of about 4.5 min. The shift of ^7^Li was referenced to 1 M LiCl in water at 0 ppm. The spectra were processed in the Bruker Topspin software using the automatic phase and baseline correction. Further data processing was done in R. The total intensity of the Li metal peak was integrated over the ^7^Li shift range of 310–220 ppm and normalized to the intensity measured at the end of plating in the first cycle.

Due to the use of metallic electrodes in the cell, the skin depth effect must be considered when NMR spectra of lithium cells are recorded. The NMR experiment relies on the ability of the used radiofrequency field to penetrate the lithium metal to a certain depth, which is called the skin depth. As the radiofrequency only penetrates the subsurface region of 12 μm in our experiment, the bulk metal signal is only proportional to the surface area and not to its volume.^5^ To detect any change in the surface area, the total metal peak (220 ppm to 310 ppm) was integrated and plotted as “total metal” in this paper. The formed microstructure during the deposition, however, is reported in the literature to be smaller than the skin depth, and therefore, the intensity of the microstructure peak is directly proportional to their mass. The quantification of the metal signals followed the theory developed and described by Bhattacharyya *et al.*^20^

## Author contributions

SM and JBF developed the concepts of soft shorts during lithium metal plating and the coupling of *operando* NMR with EIS. SM developed the *in situ* GEIS method for detection of short circuits and JBF performed and optimized the *operando* NMR experiments beyond technical aspects. RL, CdL and YC prepared electrochemical cells and assisted with NMR measurements. SM, JBF and RL processed the experimental data, performed the analysis, drafted the manuscript and designed the figures. SM, JBF, RL, ABG and CPG contributed to the interpretation of the results. All authors provided critical feedback and helped shape the research, analysis, and manuscript.

## Conflicts of interest

There are no conflicts to declare.

## Supplementary Material

FD-248-D3FD00101F-s001
