# Supplementary material for: Insights into soft short circuit-based degradation of lithium metal batteries
Source: Faraday Discuss. 2023 Jun 19;248:277–97. doi: 10.1039/d3fd00101f (PMC10823489; doi:10.1039/d3fd00101f)
Supplement: FD-248-D3FD00101F-s001 [file FD-248-D3FD00101F-s001.pdf]

Electronic Supplementary Material (ESI) for Faraday Discussions.
This journal is © The Royal Society of Chemistry 2023

Supplementary Information

Insights into Soft Short Circuit-based Degradation of Lithium Metal Batteries

Svetlana Menkin[1,2*], Jana B. Fritzke[1,2], Rebecca Larner[1], Cas de Leeuw[1,3], Yoonseong Choi[1], Anna B. Gunnarsdóttir[4], and Clare P. Grey[1,2*]

1. *Yusuf Hamied Department of Chemistry, University of Cambridge, Lensfield Road, Cambridge CB2 1EW, UK*
2. *The Faraday Institution, Quad One, Harwell Science and Innovation Campus, Didcot, U.K.*
3. *Present address: Radboud University in the Institute for Molecules and Materials (IMM), Heyendaalse weg 135 Nijmegen, Netherlands*

Contents

The effect of temperature on charge transport during Li plating	2
Galvanostatic Electrochemical impedance spectroscopy	3
The effect of current density and electrolyte composition on charge transport in the cell during polarisation cycling	5
The coupling in operando NMR and GEIS	8
Impedance analysis	11
In operando NMR	13
References	14

1. <u>The effect of temperature on charge transport during Li plating</u>

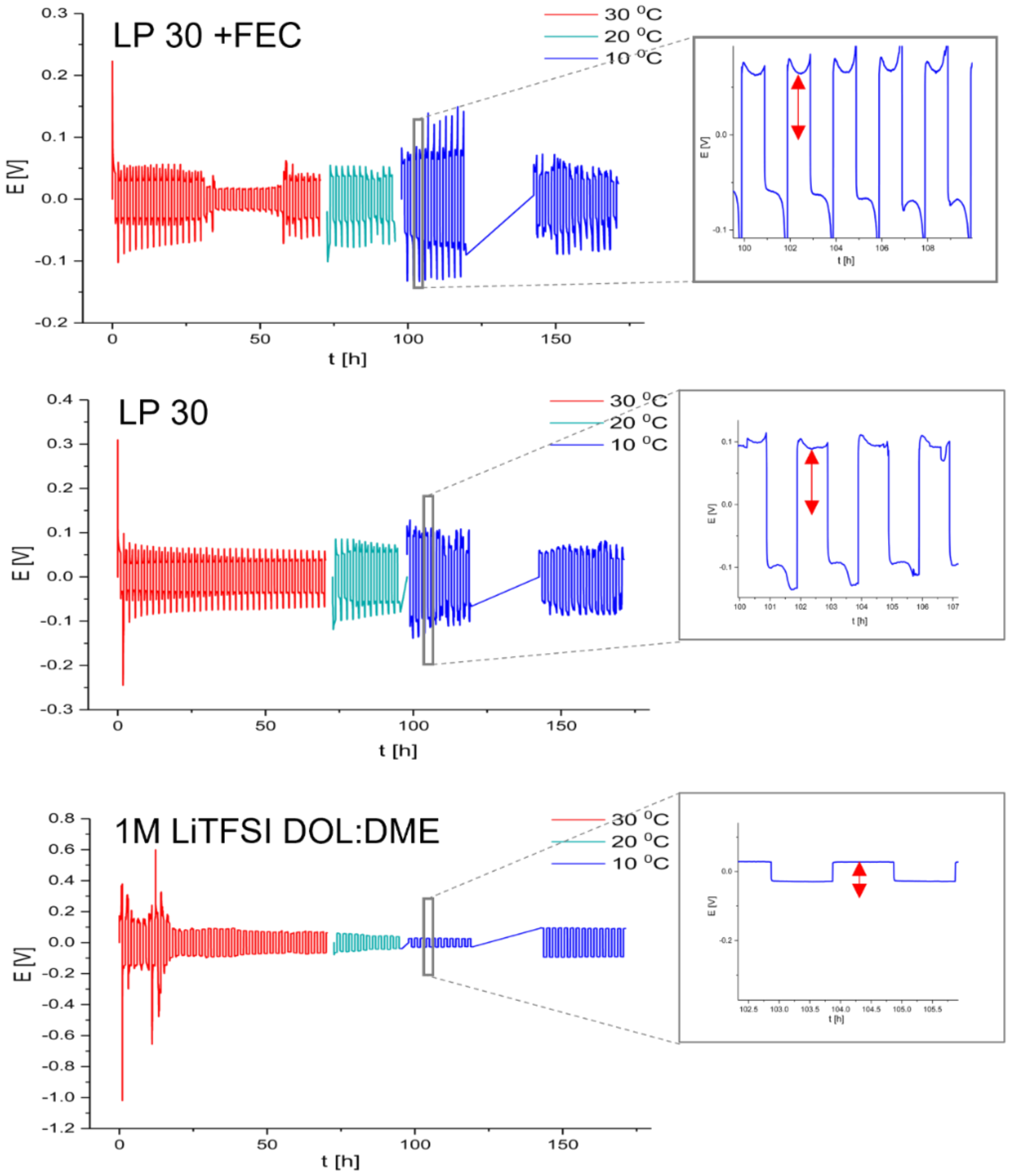

Figure S1a *Overpotential evolution with temperature in Li symmetric cells with Celgard-GF-Celgard separator. LP30 +FEC (top), LP30 (middle), and DOL:DME (bottom). Cycled at 1 mA cm*$^{-2}$ *at 30-10°C.*

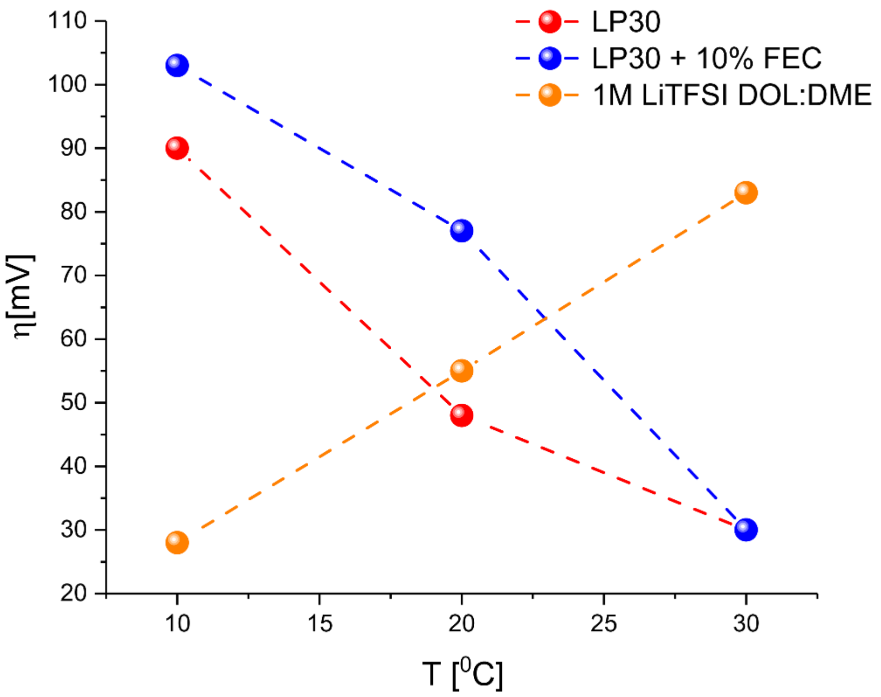

Figure S1b *Overpotential evolution with temperature in Li symmetric cells with a GF separator. LP30 (red), LP30 + FEC (blue) and DOL:DME (orange). Cycled at 1mA cm*$^{-2}$ *at 30-10°C.*

2. <u>Galvanostatic Electrochemical impedance spectroscopy</u>

Galvanostatic Electrochemical impedance spectroscopy (GEIS) is a simple and non-destructive technique which measures impedance during cycling. As such, GEIS has been used for *in-situ* impedance measurements previously for measuring the impedance of solid electrolyte-based cells.[1]

The symmetric cells are sensitive to small voltage perambulations; thus, GEIS is expected to have a lesser impact on the cell. GEIS spectra were acquired in a narrow frequency range (500-0.8 Hz), probing three points per decade. The narrower range minimises the effect on the cell since the spectrum acquisition lasts only nine seconds, and low frequencies are avoided. Indeed, the prolonged galvanostatic experiments resulted in typical voltage traces, despite the disruptions due to the GEIS (which resulted in the spikes seen on the voltage traces, see Figures 5a, 6, S4-7).

Plots of impedance intensity vs time show similar trends comparing the traces at the entire range of the measured frequencies (Figure S2). The measurements at 9 Hz were chosen to represent the GEIS measurements.

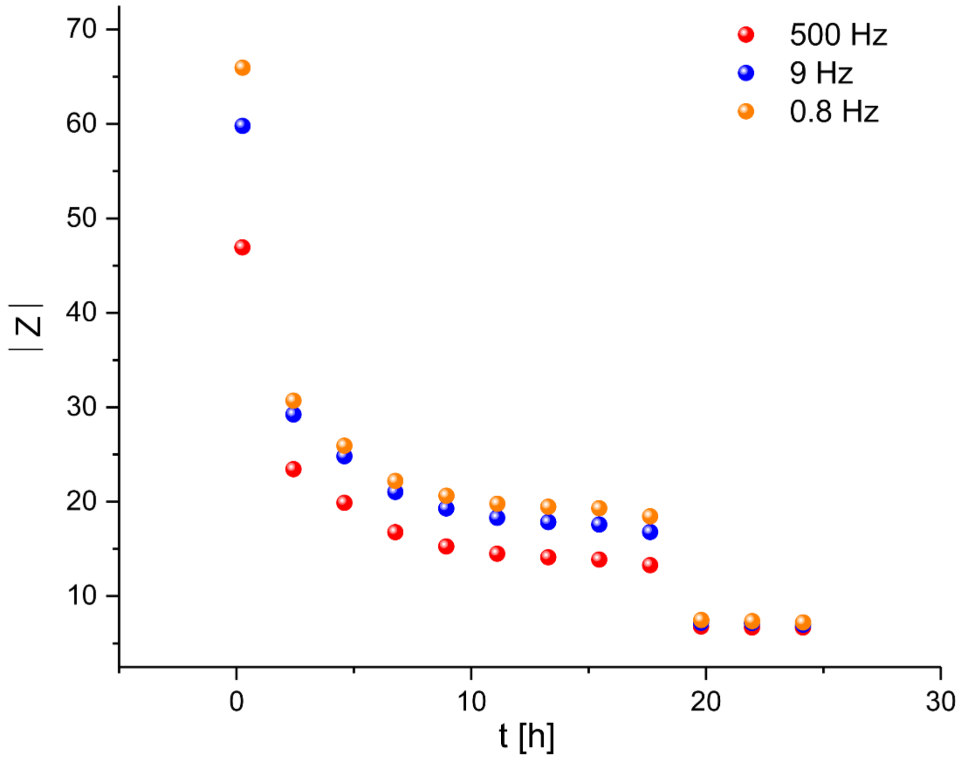

Figure S2 *Comparison of impedance intensities |Z| against at different frequencies across the range being investigated, recorded during galvanostatic plating of Li in LP30 electrolyte 1 mAcm*$^{-2}$ *in coin cell. The impedance intensity follows a similar trend in 500, 9 and 0.8 Hz.*

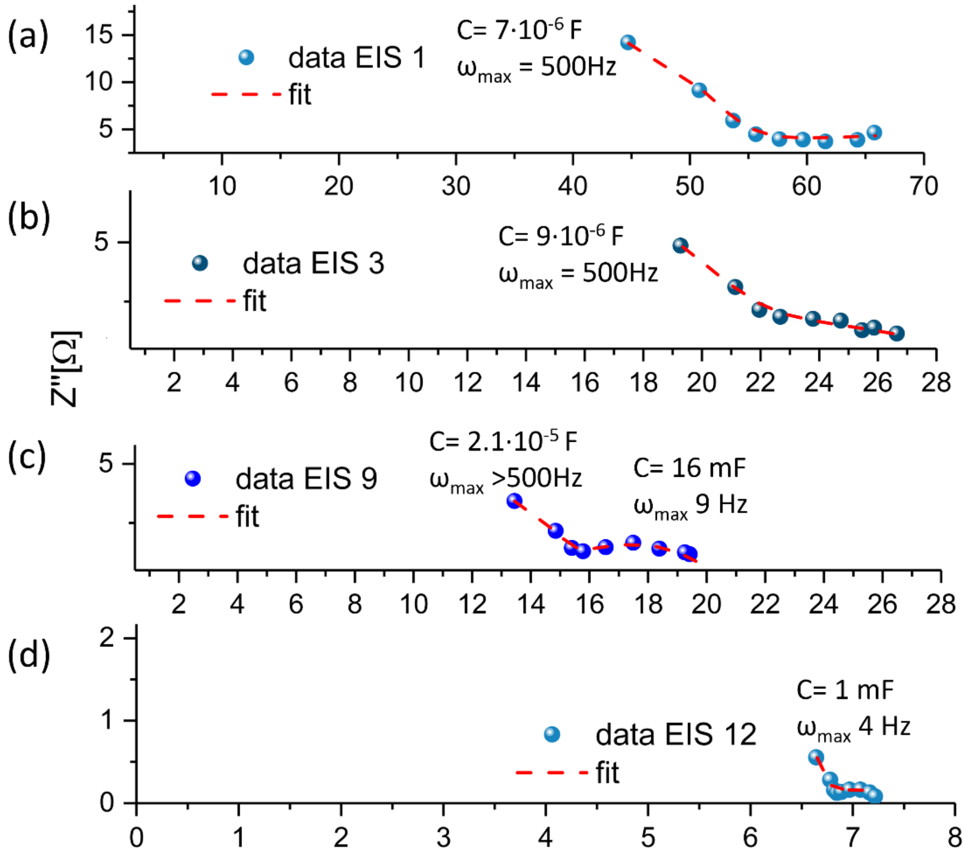

Figure S3 *GEIS Nyquist spectra (amplitude 100 µA, 500 kHz- 0.8 Hz) measured during unidirectional Li plating at 1 mA cm^{-2} in an NMR in-situ cylindrical symmetrical Li cell with LP30 electrolyte. Measured after plating approximately (a) 0.092, (b) 4.43, (c) 17.5 and (d) 24.0 mAh cm^{-2}.*

3. <u>The effect of current density and electrolyte composition on charge transport in the cell during polarisation cycling</u>

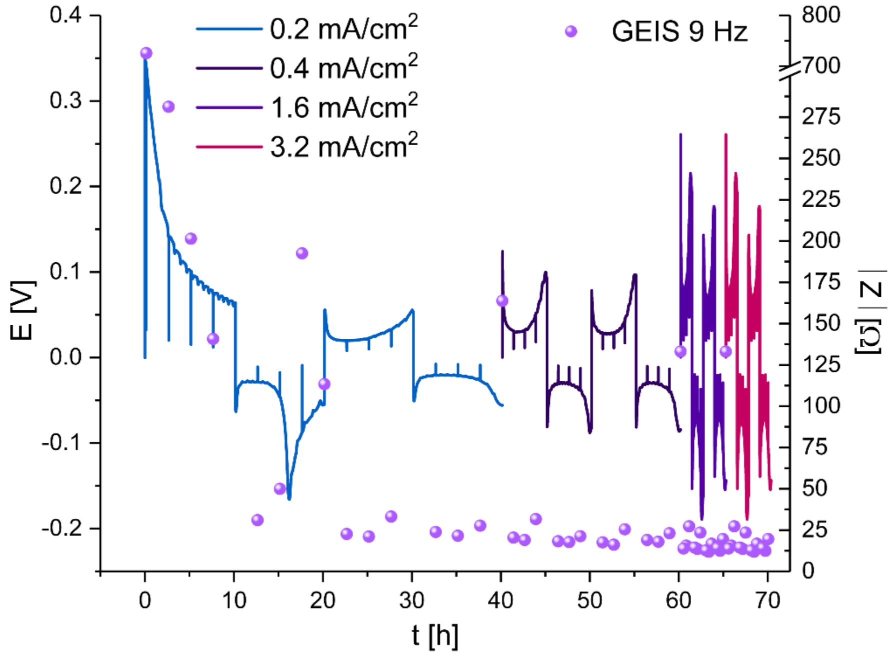

Figure S4 *Voltage-time profile of galvanostatic plating of 1mAh cm^{-2} at current densities between 0.2-3.2 mA cm^{-2} in lithium symmetric cell, LP30 electrolyte and Celgard 3501 separator at room temperature. The EIS intensity (|Z|) (purple spheres) was measured with GEIS at 9 Hz.*

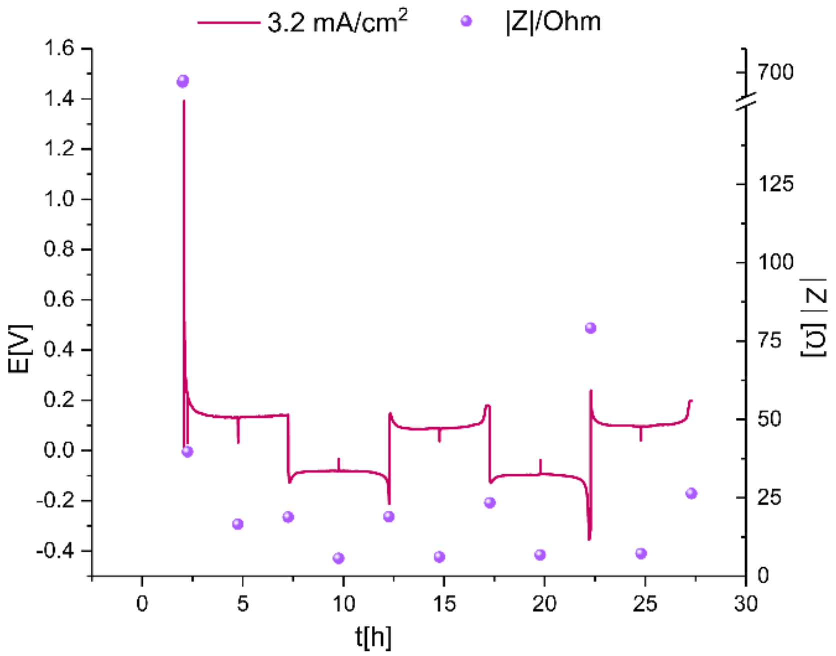

Figure S5 *Voltage-time profile of galvanostatic plating of 16 mA h cm^{-2} at 3.2 mA cm^{-2} in lithium symmetric cell, LP30 +FEC electrolyte, GF separator. EIS was measured at 10°C. GEIS intensity at 9 Hz is presented.*

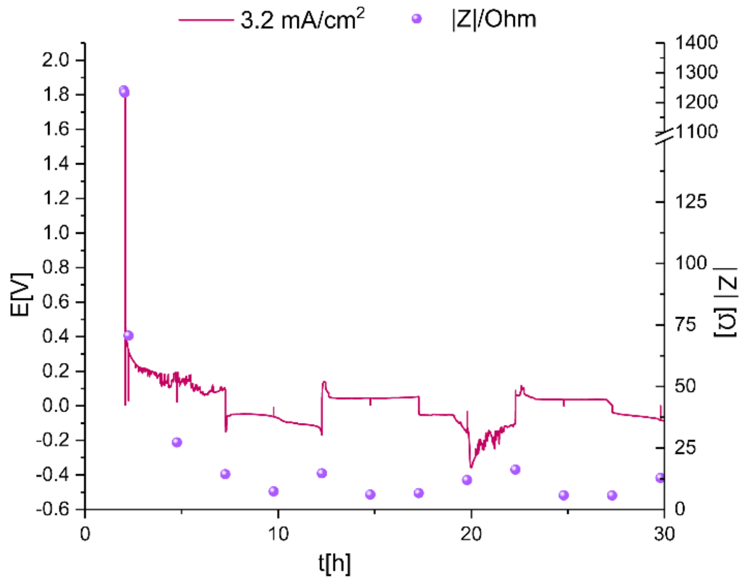

Figure S6 *Voltage-time profile of galvanostatic plating of 16 mA h cm^{-2} at 3.2 mA cm^{-2} in lithium symmetric cell, LP30 electrolyte, GF separator. EIS was measured at 10°C. GEIS intensity at 9 Hz is presented.*

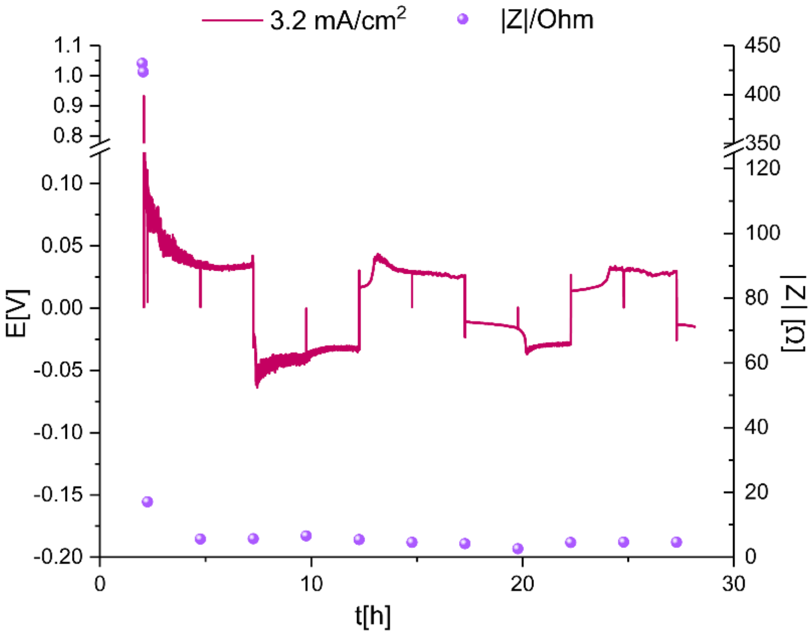

Figure S7 *Voltage-time profile of galvanostatic plating of 16 mA h cm^{-2} at 3.2 mA cm^{-2} in lithium symmetric cell, 1M LiTFSI DOL: DME 1:1 electrolyte, GF separator. EIS was measured at 10°C, GEIS intensity at 9Hz are presented.*

4. <u>The coupling in operando NMR and GEIS</u>

The coupling in operando NMR and GEIS allows the correlating NMR signals and Nyquist plots at different stated of charge.

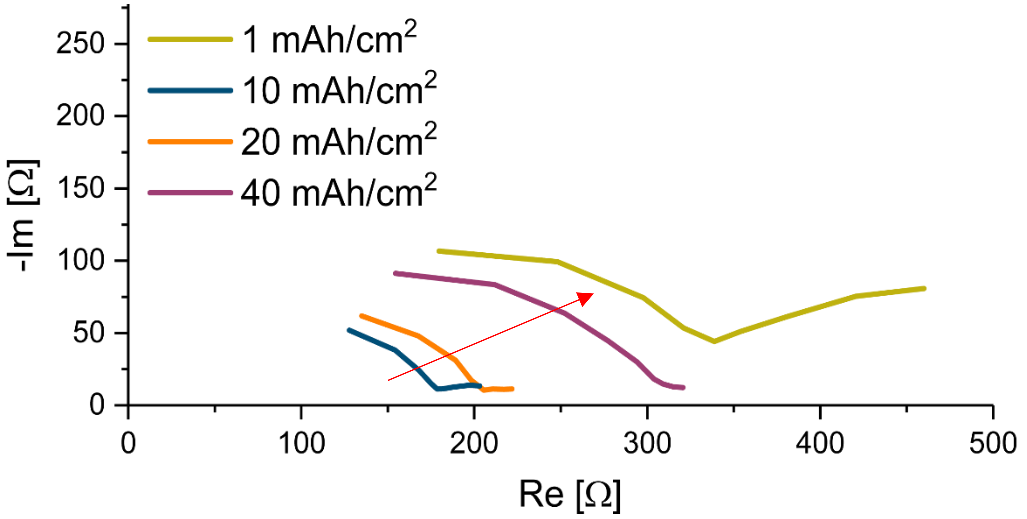

Figure S8 *GEIS Nyquist plots (amplitude 100 µA, 500 kHz- 0.8 Hz) measured during unidirectional Li plating at 0.5 mA cm^{-2} in an NMR in-situ cylindrical symmetrical Li cell with LP30 electrolyte (see Figures 7 and 8 for the operando NMR intensities).*

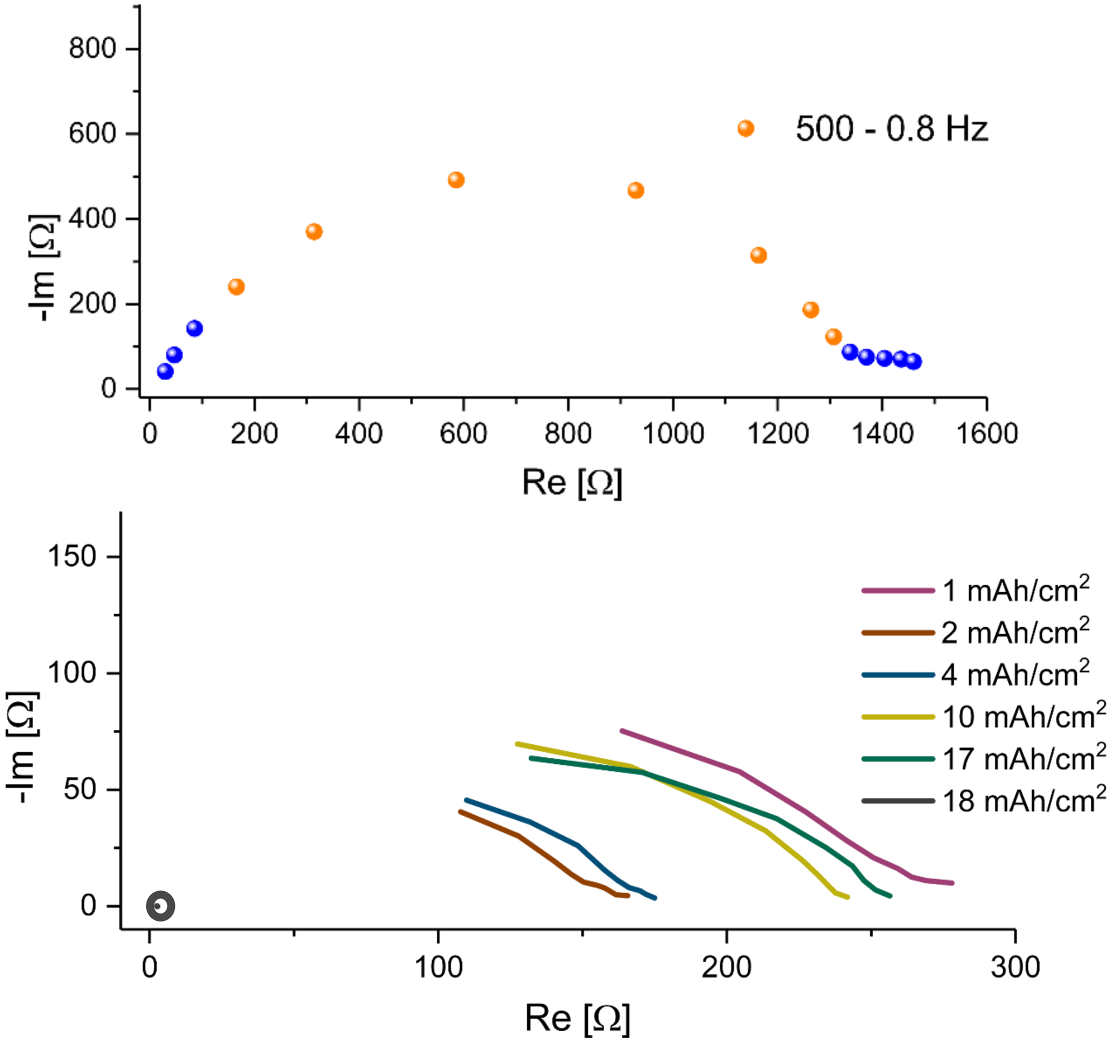

Figure S9 *GEIS Nyquist spectra (amplitude 100 µA, 500 kHz- 0.8 Hz) measured during unidirectional Li plating at 1 mA cm^{-2} in an NMR in-situ cylindrical symmetrical Li cell with LP30 electrolyte (see Figure 9 for the operando NMR intensities).*

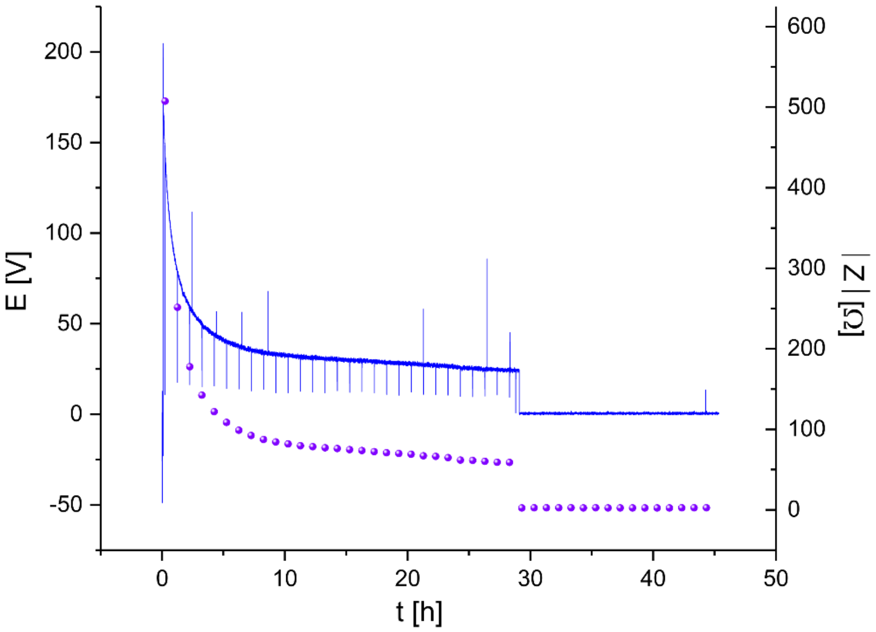

Figure S10: *Voltage traces of galvanostatic plating and GEIS intensity (at 9 Hz) measured with 100 µA amplitude at RT during Li plating at 0.5 mA cm*$^{-2}$ *in an NMR in-situ cylindrical symmetric cell, using LP30 + FEC electrolyte with GF separator.*

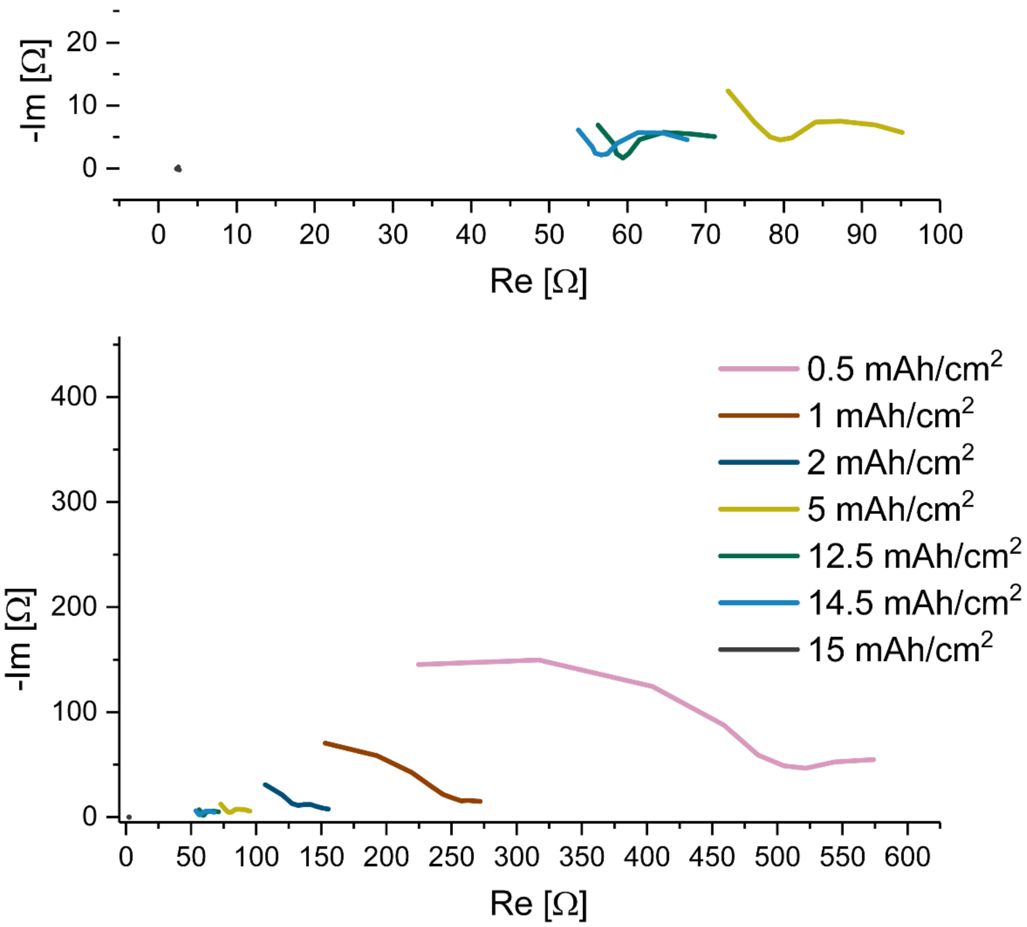

Figure S11 *GEIS Nyquist plots measured with 100 µA amplitude at RT during Li plating at 0.5 mA cm^{-2} in an NMR in-situ cylindrical symmetric cell, using LP30 + FEC electrolyte with GF separator. Magnification of the plot at 5-15 mA h cm^{-2}.*

5. Impedance analysis

To demonstrate how the nature of charge transport affects the GEIS spectra, the Nyquist plots were fitted using EC-lab software V 11.23.

The medium frequency semi-circle attributed to the SEI shifted to higher frequencies; hence, since the bulk electrolyte resistance is not likely to change, we assigned this to introducing an electronic component to charge transport in the soft shorted cell.

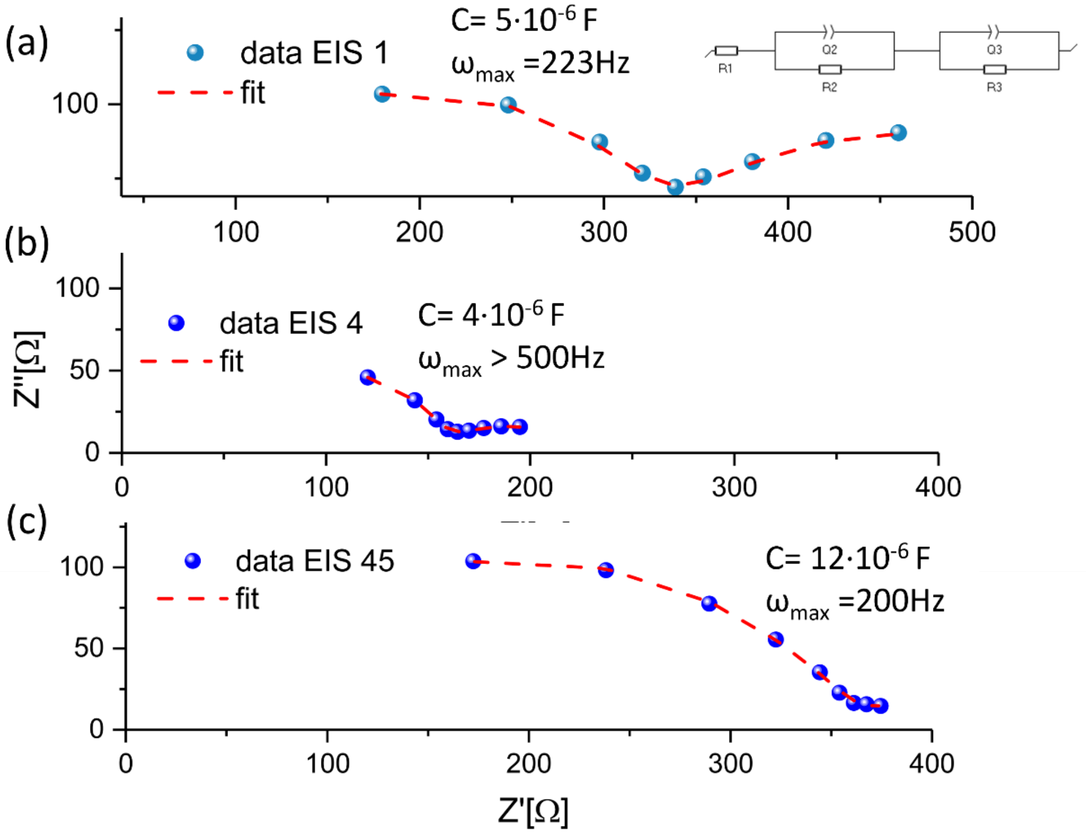

Figure S12 *GEIS Nyquist spectra (amplitude 100 μA, 500 kHz- 0.8 Hz) measured during unidirectional Li plating at 0.5 mA cm*$^{-2}$ *in an NMR in-situ cylindrical symmetrical Li cell with LP30 electrolyte. (a) measured after plating approximately 0.08* ^{2}*, (b) 1.6 and (c) 22 mAh cm*$^{-2}$ *Li.*

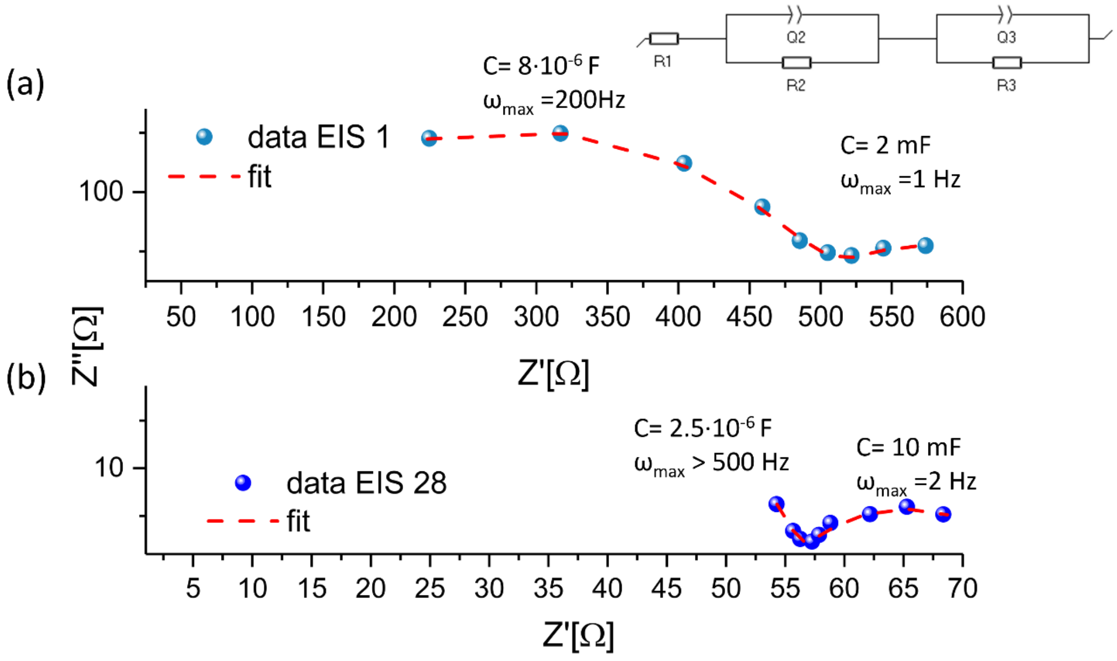

Figure S13 *GEIS Nyquist spectra (amplitude 100 µA, 500 kHz- 0.8 Hz) measured during unidirectional Li plating at 1 mA cm^{-2} in an NMR in-situ cylindrical symmetrical Li cell with LP30+FEC electrolyte. Measured after plating approximately (a) 0.08 and (b) 14.5 mAhcm^{-2} Li.*

6. *Operando* NMR

The stacked plot of the acquired ^{7}Li NMR spectra (Figure S14, right) of the Li metal plating in LP30 shows two different metal signals changing during the experiment. At the beginning of plating, only one metal signal is detectable with a chemical shift of 247 ppm. When the plating started, a second signal appeared with a chemical shift of 263 ppm, which was assigned, due to the BMS effect, to the formed microstructures on the surface of the electrode.[2] This new metal peak increases until the cell is shorted after 17 hours of plating. This short was detected with the impedance and galvanostatic measurements (Figure S14, left). After the short circuit, the shape and chemical shift of the metal signals stays constant, which indicates a permanent short circuit in the cell.

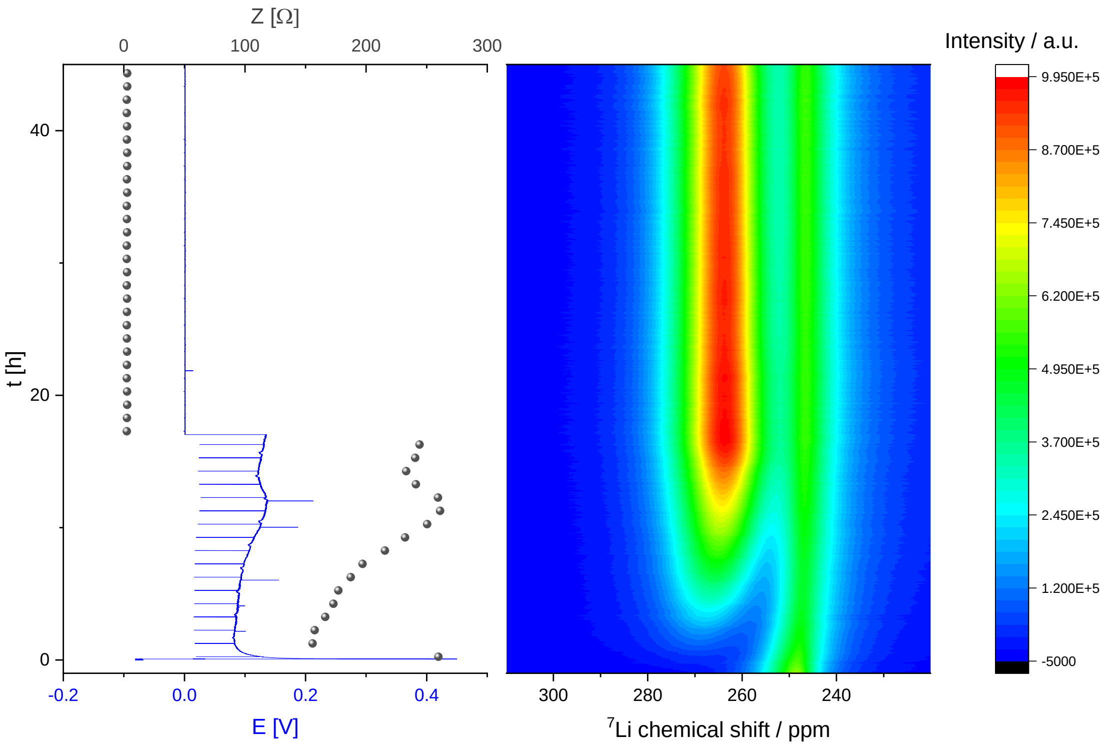

Figure S14 Operando ^{7}Li NMR spectra and impedance (GEIS) measured at 8 Hz intensities measured during unidirectional Li plating at 1 mA cm^{-2} in an NMR in situ cylindrical symmetrical Li cell with LP30 electrolyte.

Measuring a cell with LP30 + FEC shows a similar behaviour during the first 15 hours of the experiment. During steady plating, a new signal with a higher chemical shift (265 ppm) next to the bulk metal peak (246 ppm) appears.[2] The new signal increases in intensity until 15 hours, and after that, both metal peaks stay constant in intensity and shape until the end of the experiment (Figure S15, right).

This behaviour can be explained by the fact that no further plating occurs after 15 hours due to a short circuit in the cell. The voltage profile shows a normal plating curve around 15 hours, indicating a soft short degradation mechanism in the cell that prevents further Li metal plating. However, after 25 hours, the voltage and the impedance drop to zero, indicating a hard short circuit. (Figure S15, left).

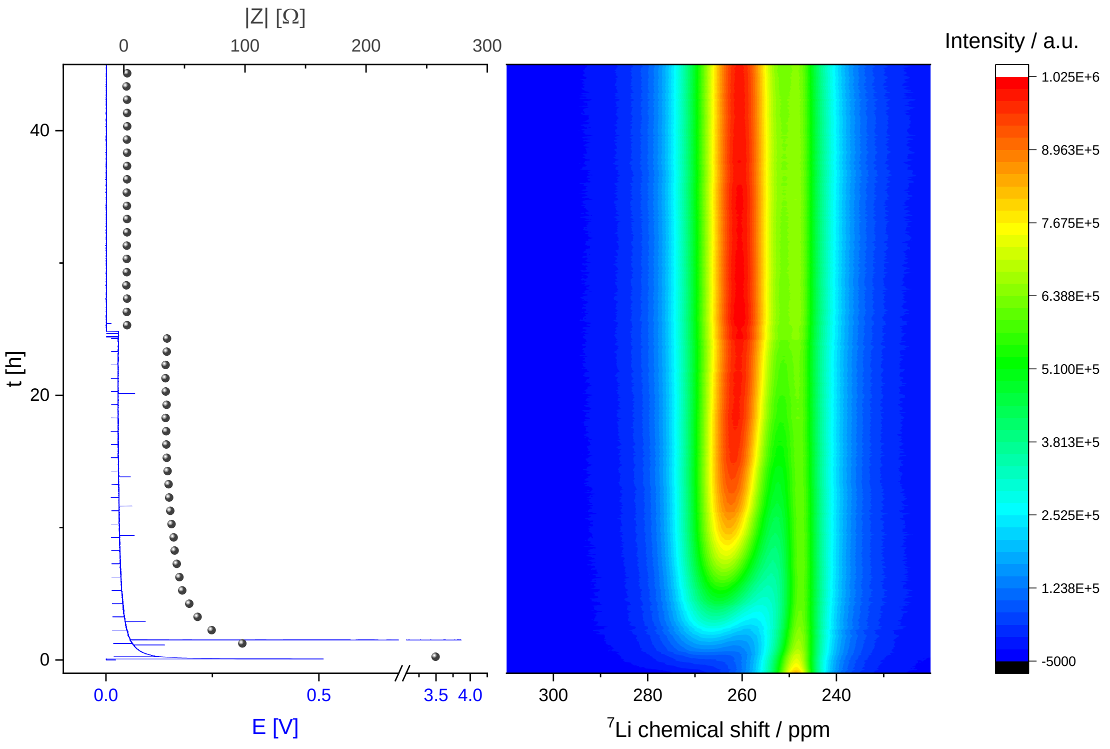

Figure S15 *Operando* ^{7}Li NMR spectra and impedance (GEIS) measured at 8 Hz intensities measured during unidirectional Li plating at 1 mA cm^{-2} in an NMR *in situ* cylindrical symmetrical Li cell with LP30 + FEC electrolyte.

<u>References</u>

1 T. Krauskopf, R. Dippel, H. Hartmann, K. Peppler, B. Mogwitz, F. H. Richter, W. G. Zeier and J. Janek, *Joule*, 2019, **3**, 2030–2049.

2 R. Bhattacharyya, B. Key, H. Chen, A. S. Best, A. F. Hollenkamp and C. P. Grey, *Nat Mater*, 2010, **9**, 504–510.
